# Rewiring of LEUNIG_HOMOLOG interaction networks marks regulatory shifts from meristem to organ growth in Arabidopsis flowers

**DOI:** 10.1111/tpj.70585

**Published:** 2025-11-24

**Authors:** Rosario Vega‐León, Jose M. Muino, Tao Zhu, Joanna A. Gluza, Maurizio Pimentel, Konstantin Rehbein, Saoirse Steiner, Emilia Rezzolla, John F. Golz, Dijun Chen, Kerstin Kaufmann, Cezary Smaczniak

**Affiliations:** ^1^ Institute for Biology, Plant Cell and Molecular Biology Humboldt‐Universität zu Berlin Berlin 10115 Germany; ^2^ State Key Laboratory of Pharmaceutical Biotechnology, School of Life Sciences Nanjing University Nanjing 210023 China; ^3^ School of BioSciences, The University of Melbourne Royal Parade Parkville Victoria 3010 Australia; ^4^ Present address: Łukasiewicz Research Network – Polish Center for Technology Development Wrocław Poland

**Keywords:** LEUNIG_HOMOLOG, LEUNIG, flower development, transcriptional regulation, Groucho/Tup1, co‐regulator, protein–protein interaction

## Abstract

LEUNIG (LUG) and LEUNIG_HOMOLOG (LUH) are Groucho/Tup1‐type transcriptional co‐regulators in *Arabidopsis thaliana* that act redundantly across multiple developmental and environmental response pathways. Their specific contributions to flower development, however, have remained unclear due to embryonic lethality of double mutants. Here, we show that LUH associates with distinct sets of transcription factors and chromatin‐associated proteins in a developmentally dynamic manner. Chromatin occupancy and protein interactions shift from a meristem‐focused network during early floral patterning to an organogenesis‐oriented state as primordia initiate and expand. Reduced promoter‐proximal LUH binding coincides with this proliferative phase, suggesting a transient reconfiguration of LUH activity. By later stages, LUH activity is amplified alongside organ differentiation programs. Together with LUG, LUH modulates gene expression programs that are essential for establishing floral organ patterning. These findings reveal how dynamic co‐regulator assemblies contribute to the temporal coordination of growth and spatial pattern formation in Arabidopsis flowers.

## Significance Statement

This study reveals that the transcriptional co‐regulator LUH interacts with distinct transcriptional and chromatin‐associated partners in a stage‐specific manner during flower development. By linking the dynamic activity of LUH to spatial patterning and organ identity, we provide new mechanistic insights into how Arabidopsis floral morphogenesis is coordinated by the LUH gene regulatory network.

## INTRODUCTION

Transcriptional co‐regulators are central mediators of either activating or repressive activities of transcription factors (TFs) in eukaryotes and are commonly conserved across kingdoms. Co‐regulators typically recruit proteins with histone‐modifying activities or possess intrinsic enzymatic activities that alter chromatin status. While mutant analyses suggest pleiotropic functions, the target‐gene specificity of co‐regulators is mediated by their interactions with a variety of TFs. Many TFs have dual functions in activating or repressing gene expression. However, the mechanisms of target‐gene‐specific recruitment of co‐regulators remain poorly understood and under investigated.

LEUNIG_HOMOLOG (LUH) is a transcriptional cofactor that belongs to the highly conserved Groucho/Tup1 protein family, which includes members found in plants, animals, and fungi. In plants, this protein family can be further classified into two subfamilies: TOPLESS (TPL)/TPL‐related (TPR) and LEUNIG (LUG)/LUH (Liu & Karmarkar, 2008). A distinctive feature of the LUG/LUH proteins is the presence of a LUFS domain at the N‐terminus, which likely contributes to their functional diversification from the TPL/TPR subfamily. The LUFS domain mediates homo‐tetramerization (Wang et al., 2019) and participates in protein–protein interactions (Sridhar et al., 2004). In addition, the central Q‐rich region and the C‐terminal WD‐repeat domain collectively contribute to the protein–protein interaction networks and regulatory functions of LUH and LUG (Lee & Golz, 2012). Since members of the Groucho/Tup1 protein family lack a distinct DNA‐binding domain, they have to be recruited by TFs to exert their transcriptional roles (Causier et al., 2012; Liu & Karmarkar, 2008; Long et al., 2006). Other co‐regulators, such as SEUSS (SEU) and its homologs SEUSS‐LIKE1‐3 (SLK1‐3), interact with both LUG and LUH (Bao et al., 2010; Franks et al., 2002; Shrestha et al., 2014; Sitaraman et al., 2008) and function as bridging adaptors that recruit TFs into LUG‐containing co‐repressor complexes (Grigorova et al., 2011; Sridhar et al., 2004, 2006), whereas TF recruitment to LUH‐containing complexes has not been demonstrated.

The functions of LUH have been explored in different contexts, including seed coat mucilage extrusion (Huang et al., 2011; Walker et al., 2011), regulation of light‐dependent seed germination (Lee et al., 2015), and tolerance to aluminum toxicity, salt, and osmotic stress (Geng et al., 2017; Shrestha et al., 2014). Additionally, LUH is implicated in the regulation of leaf polarity and meristem activity and interacts with YABBY TFs in yeast two‐hybrid assays (Stahle et al., 2009). However, its role in flower organ development remains enigmatic. In contrast, LUG plays a pivotal role in specifying floral organ identity by repressing the homeotic C‐class gene *AGAMOUS* (*AG*) and preventing ectopic expression of the B‐class genes *APETALA3* (*AP3*) and *PISTILLATA* (*PI*) in outer floral whorls (Liu & Meyerowitz, 1995). This regulation is thought to occur through cooperative interactions with the whorl‐specific MADS‐domain proteins APETALA1 (AP1) and SEPALLATA3 (SEP3) (Sridhar et al., 2006). Furthermore, LUG interacts with histone deacetylases (HDACs), facilitating its repressive mechanism through the removal of histone acetylation marks (Gonzalez et al., 2007; Sridhar et al., 2004; Tian & Chen, 2001).

Unlike *lug* mutants, *luh* mutants show no obvious floral phenotype. Homozygous *lug luh* double mutants with strong alleles are embryo‐lethal, whereas weaker allele combinations occasionally yield rare escapes with severe abnormalities in flowers and other organs (Sitaraman et al., 2008; Stahle et al., 2009). Heterozygous *lug luh*/+ mutants also show a more severe floral phenotype than *lug* mutants alone. The enhancement of *lug* mutant phenotypes by partial loss of *LUH* indicates that *LUH* and *LUG* function redundantly in flower development; however, nonredundant functions of *LUH* cannot be excluded. While *lug* single mutants do not present any shoot apical meristem (SAM) phenotypes, the *lug luh*/+ mutant displays defects in SAM patterning (Stahle et al., 2009). Moreover, LUH interacts with the MADS‐domain TFs AP1 and SEP3 in developing flowers based on the immunoprecipitation‐mass spectrometry (IP‐MS) experiments (Smaczniak, Immink, et al., 2012), and similar to LUG, is proposed to recruit HDACs to mediate transcriptional repression (Shrestha et al., 2014).

Although Groucho/Tup1 proteins are predominantly associated with transcriptional repression (Lee & Golz, 2012), the molecular functions of LUH and LUG have also been linked to transcriptional activation. LUG acts with the transcriptional co‐activator GRF1‐INTERACTING FACTOR 1 (GIF1) to promote leaf blade outgrowth and floral organ development (Zhang et al., 2019). LUH acts as a co‐activator of the TF *MYELOCYTOMATOSIS2* (*MYC2*) and interacts with MEDIATOR25 (MED25) and HISTONE ACETYLTRANSFERASE OF THE CBP FAMILY1 (HAC1), facilitating H3K9 acetylation at target loci to enhance transcription (You et al., 2019). These dual transcriptional functions highlight the complexity of LUH regulatory actions, but its specific molecular role in floral organogenesis has not been investigated.

In this study, we comprehensively dissect the LUH regulome to elucidate the dynamic nature of protein complexes, DNA‐binding profiles, and transcriptional networks throughout flower development. Our findings reveal that LUH constitutively associates with essential transcriptional co‐factors, histone modifiers, and chromatin remodeling complexes, while dynamically engaging with transcription factors, including the MADS‐domain, three‐amino‐acid loop extension (TALE), and GROWTH‐REGULATING FACTOR (GRF) proteins. Through high‐throughput analysis, we identify LUH target genes whose expression dynamics highlight critical developmental roles interconnected with key floral regulators such as AP1 and SEP3. Furthermore, a comparative transcriptome analysis between *luh* and *lug* mutants across developmental stages uncovers unique target genes, offering clarity on the distinct yet overlapping functions of LUG and LUH. Together these results advance our understanding of how LUH coordinates growth regulation with pattern formation through dynamic shifts in regulatory activity during Arabidopsis floral development.

## RESULTS

### LUH is broadly expressed at early flower developmental stages

**Figure 1:**
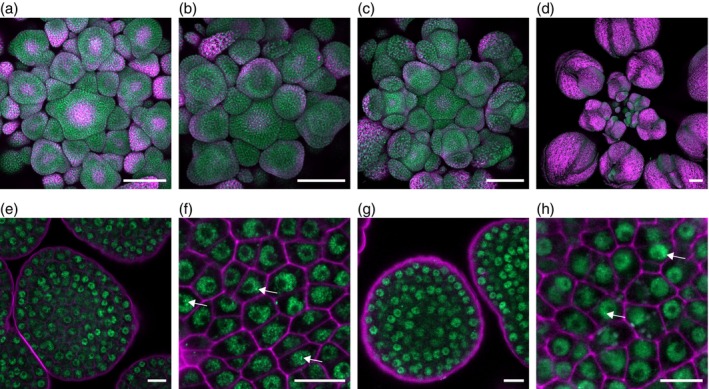
Expression pattern of *proLUH:LUH‐GFP* in FIS. GFP confocal images of inflorescences taken after induction: (a, e, f) correspond to 0 DAI; (b, g, h) correspond to 2 DAI; (c) is 4 DAI and (d) is 8 DAI. (e, g) are individual Z‐stack images of meristematic tissues taken with higher magnifications. (f, h) Show individual Z‐stack images of meristematic tissues acquired with Airyscan. Magenta signal marks chlorophyll autofluorescence in (a–d) and cell wall staining by propidium iodide in (e–h). Arrows exemplify LUH nuclear condensates. Scale bars are 100 μm in (a–d) and 10 μm in (e–h).

To characterize the spatial distribution of LUH throughout the early stages of flower development, we analyzed the GFP expression in plants containing the *proLUH:LUH‐GFP* construct (Smaczniak, Immink, et al., 2012) in the floral induction system (FIS) *proAP1:AP1‐GR ap1 cal* (Ó'Maoiléidigh et al., 2013) at different days after induction (DAI) of flowering (Figure 1) and in the Col‐0 wild type background (Figure S1). The *proLUH:LUH‐GFP* construct restored the seed mucilage phenotype in the *luh‐5* mutant to wild‐type levels in the best complementing line, confirming that the transgene is functional (Table S1). LUH‐GFP was broadly expressed at all developmental stages observed and was typically localized in the nucleus, except in cells undergoing cell division (Figure S1). We also examined the expression of the *proLUG:LUG(CDS)‐GFP* construct in the FIS for comparison. The expression of LUG‐GFP displayed, similar to LUH‐GFP, a predominant signal at the early stages of flower development (Figure S2a–d). However, unlike LUH‐GFP, the expression domain of LUG‐GFP was restricted to the inner floral region at 0 and 2 DAI. At 4 DAI, LUG‐GFP expression was additionally detected in sepals, but at 8 DAI, the signal was weak and restricted to stamens and carpels. The broader and stronger expression of LUH‐GFP compared to LUG‐GFP suggests that LUH contributes to regulatory functions during flower development independently of LUG, particularly in the outer whorls and later stages, where LUG‐GFP signal was markedly reduced. A similar broad expression pattern to LUH‐GFP was observed for the SEU‐GFP fusion protein using the *proSEU:SEU‐GFP* construct in FIS (Figure S2i–l). Importantly, LUH‐, LUG‐, and SEU‐GFP all localized to the nucleus, consistent with their roles as transcriptional co‐regulators (Figure 1e–h; Figures S1 and S2e–h,m–p). Moreover, all three proteins formed granular structures indicative of nuclear condensates (Figure 1f,h; Figures S1 and S2), compartments associated with phase separation, suggesting that they operate through large, multimeric protein assemblies. Notably, SEU has recently been shown to undergo phase separation into such condensates in Arabidopsis roots (Wang et al., 2022), in line with our observations.

### LUH interacts with key transcriptional regulatory proteins

**Figure 2:**
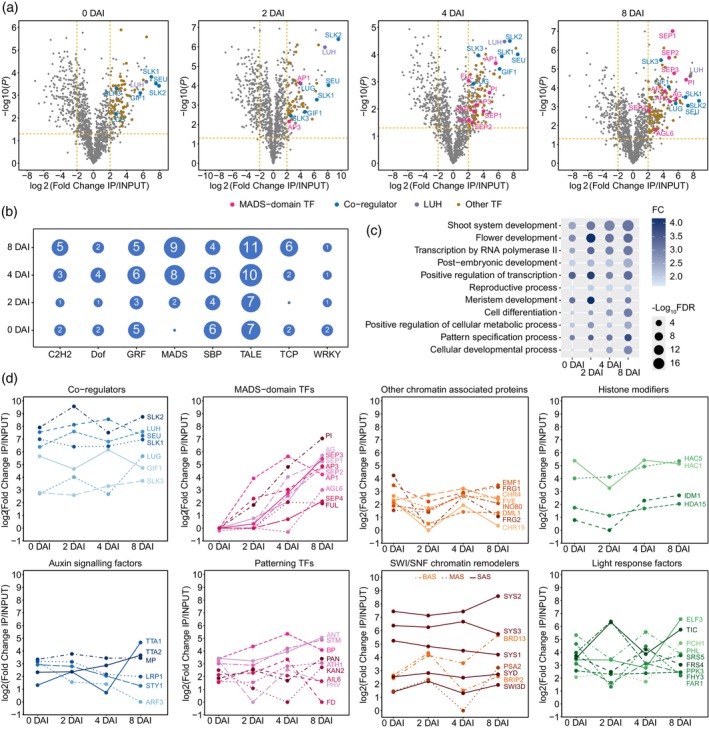
Dynamic changes of LUH protein interaction partners across different stages of flower development. (a) Volcano plots of proteomic differences between LUH‐GFP IP and INPUT samples at different DAI. Dashed lines mark thresholds: |log_2_FC| = 2 and *P* = 0.05. Only selected proteins are labeled. (b) Number of TFs belonging to specific TF families enriched in the IPs of LUH‐GFP with log_2_FC > 2 and *P* < 0.05. Only TF families that were significantly enriched (FDR < 0.05) in the GO analysis in at least a single DAI time point are listed. (C) Top GO BP annotations of the proteins enriched in the IPs. (D) Protein enrichment dynamics across different DAI for selected groups of proteins. BAS, SAS, and MAS correspond to BRAHMA (BRM)‐, SPLAYED (SYD)‐, and MINU1/2‐associated SWI/SNF complexes.

To investigate the formation of LUH protein–protein interactions at various stages of flower development, we conducted a series of IP‐MS experiments using the LUH‐GFP FIS plant line. Our analysis revealed that LUH interacted with many proteins, including various TFs (Figure 2a; Figure S3; Data S1). For further analysis, we considered only proteins with a *P* < 0.05 and a fold change (FC) > 4 in enrichment compared to the input as putative interaction partners of LUH. Using this criterion, we identified 176 proteins enriched in LUH‐GFP IPs at 0 DAI, 119 at 2 DAI, 199 at 4 DAI, and 198 at 8 DAI (Data S1). Consistent with earlier *in vitro* studies (Shrestha et al., 2014; Sitaraman et al., 2008), SEU, SLK1, and SLK2 were enriched in all IP‐MS experiments across developmental stages, validating our approach. We also detected the co‐regulators GIF1 and LUG as well as GRF TFs as potential LUH interactors (Figure 2a,b; Data S1), suggesting that they may work together within a complex. In addition, multiple MADS‐domain TFs, including AP1 and SEP3, were enriched (Figure 2a,b), extending previous reports of LUH‐MADS associations (Smaczniak, Immink, et al., 2012). Because MADS TFs are central regulators of floral development, their enrichment supports an important role for LUH in floral organogenesis. Many other TFs were enriched in the IP‐MS experiments suggesting a broader and previously underappreciated role of LUH during flower development.

Analysis of LUH‐interacting proteins revealed Gene Ontology (GO) enrichment of several other TF families, including C2H2 zinc fingers, Dof, GRF, SBP/SPL, TALE, TCP, and WRKY (Figure 2b; Data S1). Many of the identified LUH‐interacting proteins were regulators with established roles in floral development; for instance, SQUAMOSA Promoter‐Binding Protein‐Like 9 (SPL9), SPL8 and SPL15 (Wang et al., 2009; Xing et al., 2013); TALE protein SHOOT MERISTEMLESS (STM) (Endrizzi et al., 1996; Hamant & Pautot, 2010); and TCP class II TFs, TCP4, and TCP5 (Nag et al., 2009; van der Es et al., 2019). The LUH interactome also included YIN YANG 1 (YY1), a C2H2 protein that is involved in abscisic acid signaling and stress responses (Li et al., 2016). Together, these results highlight LUH as a versatile transcriptional co‐regulator in plant development, with potential links to environmental response pathways.

Further GO analysis of LUH protein interactors (Data S2) highlighted biological processes related to shoot, meristem, and flower development (Figure 2c). The top molecular function term was “Transcription regulator activity” (Data S2), consistent with the association of LUH with transcriptional control. We also found overrepresentation of liquid–liquid phase separation proteins (Data S2), such as EARLY FLOWERING 3 (ELF3), which has been shown to phase separate in Arabidopsis (Hutin et al., 2023). This enrichment is in line with our confocal observations.

Next, we examined the dynamics of LUH interactions with selected functionally related protein groups. In addition to TF families and other co‐regulators, LUH interacted with chromatin remodeling and chromatin‐associated proteins, histone modifiers, auxin signaling proteins, light response factors, and other organ patterning TFs (Figure 2d). Some LUH partners were constitutively present, interacting with LUH at every developmental time point. Examples include histone acetylases HAC1 and HAC5, which act as transcriptional activators by deposition of acetylation marks. Their interaction with LUH is consistent with a previously reported indirect *in vitro* interaction (You et al., 2019). Other stable LUH partners comprised chromatin remodeling and chromatin‐associated proteins, suggesting that LUH may influence chromatin organization. In contrast, certain interactions were dynamic, such as those with MADS‐domain TFs, which associated more abundantly with LUH at later stages. These dynamic changes in protein enrichment were not explained by transcript levels of the corresponding genes (Figure S4), indicating genuine shifts in LUH protein complexes during flower development rather than indirect effects of expression.

### LUH bridges interactions between TFs and chromatin regulators

**Figure 3:**
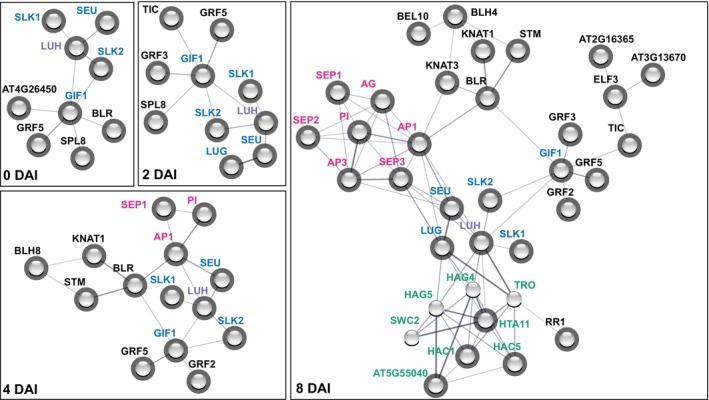
Protein interaction networks of LUH at different stages of flower development. LUH IP‐MS enriched interactors at specific DAI with a more stringent cutoff of log_2_FC > 4 and *P* < 0.05 were used to plot the protein–protein interaction network in the STRING database. Shaded spheres correspond to LUH interactors detected with IP‐MS experiments and the STRING database; unshaded spheres correspond to STRING‐predicted interactors. Proteins that did not show any connection to LUH were removed from the network.

To confirm and predict other potential interaction partners of LUH, we drew protein interaction networks based on the data deposited in the STRING database (Szklarczyk et al., 2023). The resulting networks revealed several interconnected protein hubs, especially at 8 DAI. Importantly, the core of the network was formed by co‐regulators, including LUH and LUG, along with the bridge proteins SEU and SLK1‐2. This core hub was directly linked to the MADS‐domain TF, histone modifier, and growth regulation factor hubs, and indirectly linked to the TALE TF hub (Figure 3). The physical interaction network at 8 DAI uncovered a hub of regulators with predominantly transcriptional activating functions that are connected with LUH and LUG. This hub also included predicted interactors not detected in the IP‐MS experiments, GNAT/MYST histone acetyltransferases (HAG4 and HAG5), the chromatin remodeler SWC2, and the methyltransferase TRAUCO (TRO), which is responsible for H3K4 trimethylation. The analysis of the tissue‐specific gene expression database, the Transcriptome Variation Analysis database (TraVA DB) (Klepikova et al., 2016), revealed that *HAG4*, *HAG5*, and *SWC2* are co‐expressed with *LUH*, *LUG* and *SEU* in young flowers, carpels, and ovules while *TRO* showed weak co‐expression in carpels and ovules (Figure S5), thereby supporting the notion that they form a complex with LUH.

### LUH interacts directly with MADS‐domain TFs *in vitro*

**Figure 4:**
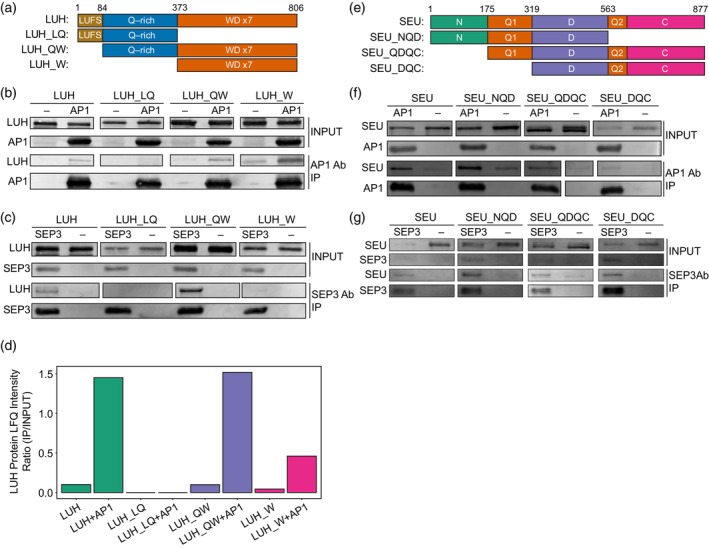
Co‐IPs of LUH and SEU reveal direct interaction with MADS‐domain TFs. (a) LUH protein truncations tested in the Co‐IPs. Domain structure is essentially as reported previously (Sitaraman et al., 2008). Numbers represent amino acid positions. (b) Co‐IPs of different LUH truncations with and without AP1 protein. (c) Co‐IPs of different LUH truncations with and without SEP3 protein. (d) Co‐IP‐MS label‐free quantification (LFQ) analysis of LUH protein intensities in the eluates and input samples from (b). Associated LFQ values are listed in Table S2. (e) SEU protein truncations tested in the Co‐IPs. Domain structure is essentially as reported previously (Sridhar et al., 2004). Numbers represent amino acid positions. (f) Co‐IPs of different SEU truncations with and without AP1 protein. (g) Co‐IPs of different SEU truncations with and without SEP3 protein. The *in vitro* expressions of the truncated LUH and SEU proteins are visualized in Figure S6. The raw Western blot acquisition scans are visualized in Figures S7 and S8.

To gain insight into the structural nature of the co‐regulator‐MADS TF protein interactions, we performed a series of *in vitro* co‐immunoprecipitation (Co‐IP) experiments. We used various LUH and SEU protein constructs comprising different domains of the proteins and assessed their ability to interact with AP1 and SEP3 (Figure 4). We confirmed that a full‐length SEU protein interacted with AP1 and SEP3, but LUH also showed a direct interaction. The LUH_W domain, which contains seven WD repeats, is sufficient for interaction with AP1, whereas interaction with SEP3 also requires the intrinsically disordered LUH_Q domain (Figure 4b,c). In contrast, LUH‐LQ, which contains the LUFS domain responsible for the direct interaction with SEU‐like proteins (Grigorova et al., 2011; Shrestha et al., 2014; Sitaraman et al., 2008), was unable to interact with AP1 or SEP3. Our results suggest that AP1 and SEP3 proteins interacted with LUH via its most C‐terminal domains (Q and WD), thus different from SEU, SLK1, and SLK2. To confirm LUH Co‐IP results, we performed LC–MS/MS quantitative analysis of the inputs and eluates from the LUH‐AP1 co‐immunoprecipitation experiments (Figure 4d; Table S2). In sum, the results showed that LUH interacts with MADS‐domain TFs and SEU via alternative protein domains, potentially enabling ternary complex formation, as previously demonstrated for LUG (Gregis et al., 2006; Sridhar et al., 2006).

Previous yeast‐two‐hybrid experiments revealed direct interactions of SEU with AP1 and SEP3, mediated via the C‐termini of the MADS domain proteins (Sridhar et al., 2006) that contain Q‐rich intrinsically disordered‐like transcriptional activation domains. Our Co‐IP data showed that SEU is bound to AP1 and SEP3 with any of its domain configurations, but most strongly with the SEU_NQD domains (Figure 4f,g). Two Q‐rich regions are found in SEU, and Q1 induces condensate formation in response to molecular crowding (Wang et al., 2022). This opens the possibility that interactions between MADS‐domain C‐termini and SEU involving their Q‐rich domains dynamically modulate the transcriptional activation potential of these TFs via phase separation‐based mechanisms.

### LUH complexes target a broad network of flower developmental genes

To assess the LUH target network during flower development, we performed chromatin immunoprecipitation followed by sequencing (ChIP‐seq) at four developmental stages (0, 2, 4, and 8 DAI). We identified a significant number of LUH‐bound regions (Table S3; Data S3), associated with both overlapping and stage‐specific target genes (Figure S9a). GO enrichment analyses revealed that LUH targets are consistently linked with developmental processes (Figure S9b; Data S4). At 0 DAI, LUH bound genes involved in meristem maintenance, such as *CLAVATA 1* (*CLV1*), *JAGGED LATERAL ORGANS* (*JLO*), *MYB87*, and *PLETHORA 1* (*PLT1*). At 2 DAI, LUH targeted *STM*, *SHEPHERD* (*SHD*), *TERMINAL FLOWER 1* (*TFL1*), and *AGAMOUS‐LIKE 19* (*AGL19*), known for regulating meristem size and flowering onset. As development progressed, LUH binding shifted toward genes involved in organ specification and differentiation, including *RABBIT EARS* (*RBE*), *AT‐HOOK MOTIF NUCLEAR LOCALIZED PROTEIN 21* (*AHL21*), *SEP3*, and *KNUCKLES* (*KNU*) at 4 and 8 DAI (Data S3). Notably, we also found LUH binding at *RESPONSIVE TO DESICCATION 20* (*RD20*), *MYB2*, and *NAC019*, stress response genes previously reported as differentially expressed in *luh* mutants (Shrestha et al., 2014), further supporting their direct regulation by LUH. This suggests that LUH dynamically targets distinct gene sets aligned with developmental transitions.

**Figure 5:**
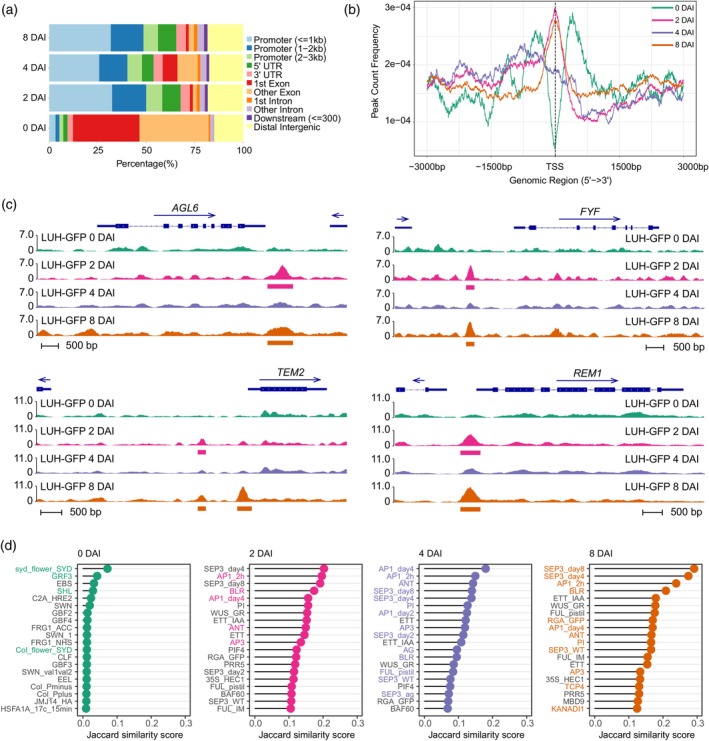
DNA binding dynamics of LUH by ChIP‐seq during different stages of flower development. (a) LUH DNA binding peak summit distribution across the genome. (b) LUH DNA binding peak count frequency in relation to gene TSS across the genome. (c) Stage‐specific binding peaks in selected, differentially expressed genes. Horizontal bars under the peaks indicate peaks called with *P* < 0.05. Arrows indicate gene directionality. (d) Similarity of LUH ChIP‐seq experiments with other published ChIP‐seq data. Jaccard similarity scores indicate the degree of overlap between LUH‐bound regions and other TF ChIP‐seq datasets (0 = no overlap, 1 = complete overlap). For context, comparisons with all available TF ChIP‐seq datasets in ChIP‐hub (Fu et al., 2022) are provided in Data S5. Colored names of ChIP‐seq experiments indicate that the TF was also stage‐specifically enriched in the LUH‐GFP IP‐MS experiments.

The majority of LUH ChIP‐seq peaks at 2, 4, and 8 DAI localized to regulatory regions (promoters, UTRs, introns), while at 0 DAI, peaks were predominantly located in exons (Figure 5a). Peak localization analysis showed enrichment near transcription start sites (TSS) at 2 and 8 DAI, whereas peaks were more upstream at 4 DAI and depleted at the TSS at 0 DAI (Figure 5b). LUH occupancy was also lower at 0 and 4 DAI compared to 2 and 8 DAI (Figure S9c). This atypical distribution at 0 DAI may reflect either technical limitations of LUH ChIP in early meristematic tissue or an alternative binding mode of LUH, and should be interpreted with caution. By contrast, the reduced LUH occupancy observed at 4 DAI was consistent with our RNA‐seq data, which revealed fewer differentially expressed genes (DEGs) at this stage compared to 2 and 8 DAI (Table S4). Visualization of ChIP‐seq data at selected, DEG loci, for example, *FOREVER YOUNG FLOWER* (*FYF*), *AGAMOUS‐LIKE 6* (*AGL6*), *REPRODUCTIVE MERISTEM 1* (*REM1*), and *TEMPRANILLO 2* (*TEM2*) highlighted this dynamic behavior (Figure 5c). These findings indicate that the DNA‐binding activity of LUH varies with developmental stage, with reduced occupancy correlating with high‐proliferation stages like organ initiation, when LUH may be displaced during cell division (Figure S1).

Comparison with public TF ChIP‐seq data from the ChIP‐Hub platform (Fu et al., 2022) revealed significant overlap with LUH targets (Figure 5d; Data S5). Specific examples include similar binding with SYD, GRF3, and EARLY BOLTING IN SHORT DAYS (EBS) at 0 DAI; SEP3, AP1, and BELLRINGER (BLR) at 2 and 8 DAI; and AINTEGUMENTA (ANT), AP1, and SEP3 at 4 DAI. Notably, many of these TFs also emerged as LUH interaction partners in our IP‐MS data, suggesting functional cooperation. Motif analysis using MEME‐ChIP supported these associations, revealing enriched DNA motifs corresponding to TF families identified in LUH protein complexes (Figure S9d). To further explore shared regulatory activity, we performed ChIP‐seq in bulk inflorescences expressing SEU‐GFP (*proSEU:SEU‐GFP seu‐1*). The strongest peak overlap was with LUH at 8 DAI, consistent with the developmental bias of the non‐staged tissue (Table S5). SEU‐bound genes showed GO enrichments similar to those of LUH targets, and we identified several common target genes, including key developmental regulators (Data S6). Together, these results highlight LUH as an important and dynamically engaged factor of the floral gene regulatory network, acting through stage‐specific DNA binding and TF‐mediated co‐regulatory complexes.

### Common and specific target gene programs of LUH and LUG in flower development

**Figure 6:**
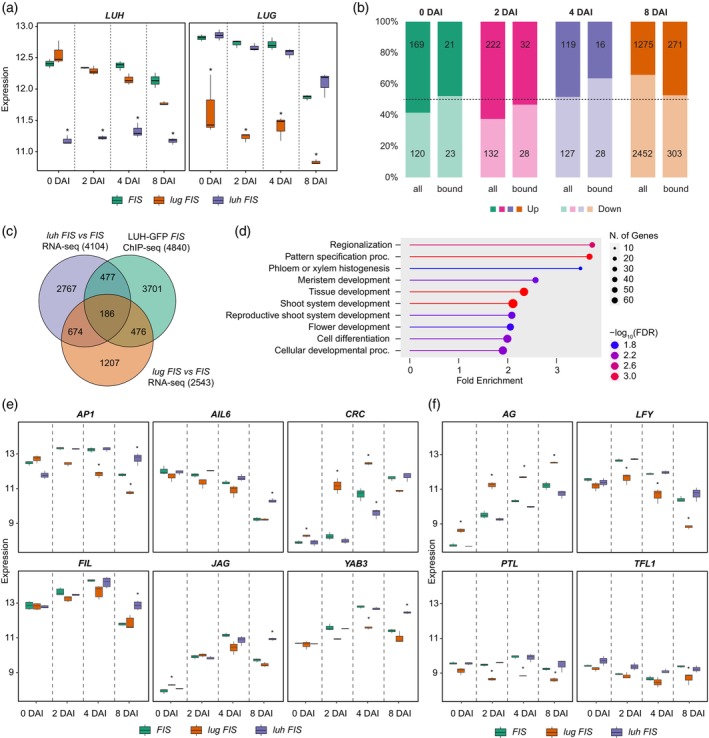
Gene expression analysis of *luh* FIS and *lug* FIS mutants during different stages of flower development. (a) Expression of *LUH* and *LUG* genes in the studied plant lines. (b) Numbers of up‐ and downregulated genes in *luh* FIS versus FIS (all) and in LUH‐bound genes at any stage (bound). (c) Venn diagram of DEGs (FDR <0.05 and |log_2_FC| > 1) in *luh* FIS versus FIS and in *lug* FIS versus FIS as well as LUH ChIP‐seq target genes (*P* < 0.05) that were significantly enriched in at least one developmental time point. Only genes closest (3 kb distance) to a significant peak (*P* < 0.05) were considered. (d) GO enrichment for the differentially expressed LUH target genes (663) using the GO BP database. Top 10 most enriched pathways are shown. For the full list of genes and detailed GO annotations, see Data S7. (e) Expression of selected *luh* FIS DEGs related to “Flower development” and “Regionalization” GO categories in different plant lines. (f) Expression of selected *lug* FIS DEGs bound by LUH related to “Flower development” and “Regionalization” GO categories in different plant lines. Expression values on the *y*‐axis in (a, e, f) represent DESeq2‐normalized counts (log_2_‐transformed, variance‐stabilized counts). Asterisks in (a, e, f) indicate DEGs (FDR < 0.05 and |log_2_FC| > 1).

To identify stage‐specific target genes of LUH and LUG, we performed transcriptomic analyses of *lug* FIS and *luh* FIS mutants at different time points of flower development. While *luh* FIS mutants showed no obvious morphological phenotype, *lug* FIS plants displayed early development of anthers and carpels as well as partial transformation of sepals into stamens and carpels (Figure S10). The mRNA‐seq confirmed reduced *LUH* and *LUG* expression in their respective mutants (Figure 6a), and replicate correlation analysis verified the high reproducibility of the RNA‐seq data (Figure S11). Despite the absence of visible phenotypes in *luh* FIS, differential expression analysis revealed changes in DEG numbers across developmental time points (Table S4; Data S8). At the earlier stages (0 and 2 DAI), *luh* FIS showed mostly upregulated genes, shifting toward downregulation at later stages (4 and 8 DAI). In *lug* FIS, DEG numbers were higher (except at 8 DAI), with a consistent bias toward gene upregulation (Table S4; Data S8). The substantial number of DEGs in *luh* FIS indicates that *LUH* broadly influences gene expression even in the absence of apparent morphological effects, likely due to redundancy with *LUG* that buffers transcriptional changes at the phenotypic level.

To pinpoint direct LUH targets, we intersected stage‐specific DEGs from *luh* FIS with LUH ChIP‐seq bound genes from all stages (Figure 6b). Notably, LUH exhibited the highest ChIP occupancy and DEG overlap at 8 DAI, indicating strong regulatory activity, primarily activating, during floral organ differentiation. Many LUH‐bound genes that were differentially expressed in *luh* FIS were not affected in *lug* FIS, suggesting distinct regulatory roles. GO analysis of LUH‐bound DEGs highlighted functions in development and spatial patterning (Figure 6c,d; Data S7). Expression of selected genes from these categories showed distinct regulation across backgrounds (Figure 6e,f). For example, the carpel regulator *CRABS CLAW* (*CRC*) was upregulated in *lug* FIS but downregulated in *luh* FIS at specific stages. *AG*, a master floral regulator, was misregulated only in *lug* FIS, which is possibly linked to precocious reproductive organ formation. Conversely, organ polarity gene *FILAMENTOUS FLOWER* (*FIL*) was upregulated in *luh* FIS at 8 DAI, while its paralog *YABBY3* (*YAB3*) showed opposing patterns in the two mutants. These distinct patterns suggest that LUH and LUG exert both independent and coordinated regulatory effects, particularly in early flower stages and inner whorl organ development.

We next analyzed expression changes in genes co‐bound by LUH and the top 10 TFs with the highest ChIP‐seq target gene similarity (Fu et al., 2022) as well as transcriptional co‐regulators GIF1 (Vercruyssen et al., 2014) and SEU. These co‐bound genes showed different dynamics than the global *luh* FIS expression profile (Figure S12; Data S9). At 8 DAI, most LUH‐TF co‐bound genes were upregulated, consistent with LUH acting as a repressor at late stages. Similarly, GIF1‐ and SEU‐LUH common targets showed little change at 2 DAI but a modest bias toward upregulation at 8 DAI in the *luh* FIS mutant (Figure S12; Data S9). This pattern aligns with the established role of SEU as a transcriptional corepressor in floral development, and suggests that SEU‐LUH complexes contribute to repression particularly at later stages. At earlier stages, co‐bound gene numbers were lower, with less consistent expression patterns. This may reflect a shift in LUH activity from attenuated, possibly more transient binding in proliferative meristem phases, to more stable repression during organ specification. Together, our data demonstrate that LUH and LUG regulate both shared and unique gene networks during flower development. LUH exhibits dynamic, stage‐specific regulatory behavior, with distinct mechanisms from LUG. These findings highlight the complex role of LUH in shaping floral gene expression programs through both direct DNA binding and TF‐mediated co‐regulatory interactions.

## DISCUSSION

Groucho/Tup1‐type co‐regulators are conserved transcriptional repressors that fine‐tune gene expression through multiprotein complexes formed with diverse transcription factors (TFs). In Arabidopsis, LUH exemplifies this versatility, coordinating dynamic and stage‐specific transcriptional programs during flower development. In this study, we uncover a previously unappreciated level of complexity in LUH function. Using *in vivo* protein complex profiling by mass spectrometry, we reveal that LUH engages in highly dynamic interactions with a broad array of TFs, including MADS‐domain, TALE, and GRF family proteins, as well as in more stable interactions with chromatin‐associated factors. These interactions change over time and developmental stage, highlighting the role of LUH as a versatile scaffold within floral co‐regulatory networks. Notably, we identify numerous novel LUH interaction partners, indicating its broader and more integrative function in transcriptional regulation than previously recognized.

The *lug luh*/+ mutant displays severe floral defects: carpelloid sepals, malformed sepal‐like organs, absent petals, and fused or missing stamens (Sitaraman et al., 2008); demonstrating an essential role for LUH in patterning the floral meristem and establishing the ground plan. Compared to LUG, LUH shows broader and more persistent expression, particularly in late flower development, which aligns with our transcriptomic data showing LUH and LUG regulate overlapping but stage‐specific gene sets. LUH target genes are enriched for regulators of meristem activity and organ polarity, supporting its function in spatial patterning and organ differentiation. LUH activity undergoes a striking developmental switch: from a meristem‐associated configuration to an organogenetic regulatory state. This transition is marked by broad shifts in its interaction landscape and a transient reduction in DNA‐binding occupancy, suggesting a reconfiguration of LUH's regulatory target networks as floral meristems initiate organ outgrowth.

LUH also appears to contribute to the regulation of SAM maintenance. While *lug* single mutants do not exhibit SAM defects, *lug luh*/+ mutants show enlarged meristems and disrupted L2 layer cell division (Stahle et al., 2009). By contrast, loss of the central SAM regulator *STM* results in reduced or absent meristems (Endrizzi et al., 1996). Additional complexity is introduced by *seu slk2* mutants, which exhibit an *stm*‐like phenotype associated with decreased *STM* expression (Lee et al., 2014), suggesting that different co‐regulators can influence *STM* through distinct mechanisms. Our findings that LUH directly interacts with STM and binds to the *STM* locus, provide a mechanistic link between LUH‐containing complexes and *STM* function. Since STM also activates key floral regulators such as *LEAFY* (*LFY*) and *AP1* (Roth et al., 2018), the LUH–STM interaction offers a direct route by which LUH connects meristem regulation with floral specification. LUH was consistently associated with GRFs and their co‐activator GIF1 in our IP‐MS experiments, both of which promote cell proliferation (Jun et al., 2019; Kim & Kende, 2004; Zhang et al., 2019). Comparison of LUH‐bound genes with published GRF and GIF1 genomic binding datasets (from non‐floral tissues) (Piya et al., 2020; Vercruyssen et al., 2014) revealed measurable overlap, and GRF binding motifs are enriched at LUH‐bound regions. These findings suggest that LUH may modulate GRF‐GIF1 activity to balance proliferation and patterning during floral organogenesis. The association of LUH with light‐response regulators, such as PIF1 (Lee et al., 2015), suggests LUH may integrate environmental signals, such as light, into developmental transcriptional programs.

Although LUH is structurally classified as a transcriptional co‐repressor (Conner & Liu, 2000; Liu & Karmarkar, 2008), it interacts with both repressors and activators. LUH associates with Mediator complex subunits, which are capable of facilitating both gene repression and activation (Chen et al., 2022). It also binds to dual‐function TFs like YY1 (Li et al., 2016) and to histone acetyltransferases HAC1 and HAC5. This supports previous findings that LUH can modulate chromatin states, including the deposition of activating histone marks (You et al., 2019). However, LUH‐TF co‐binding is predominantly associated with gene repression, suggesting that LUH may dampen the activity of activators when embedded in larger regulatory complexes.

We also find that LUH directly binds several MADS‐domain TFs such as AP1 and SEP3, with interaction levels increasing over time. These protein–protein interactions are mediated by LUH's WD‐repeat domain and occur independently of SEU. Although SEU also interacts with these TFs, its role may be limited to facilitating their recruitment into LUH‐containing complexes. Co‐binding of LUH and SEU to the same genomic loci does not enhance transcriptional activation, reinforcing the function of LUH as a repressor, even when operating in multi‐protein assemblies. LUG was identified as a complex partner of LUH, and both are recruited to regulatory regions of *AG*, a central floral homeotic gene (Conner & Liu, 2000; Liu & Meyerowitz, 1995). LUH binds the same intragenic region as LUG and other known *AG* regulators, such as BLR, PERIANTHIA (PAN), and ANT (Bencivenga et al., 2016; Das et al., 2009; Krizek et al., 2020). These factors may function as either activators or repressors depending on floral whorl context and developmental timing, and they interact with LUH based on our IP‐MS data. These observations suggest that LUH may integrate multiple regulatory inputs, potentially in a modulatory or indirect manner at the *AG* locus, while contributing more broadly to spatiotemporal control of its target genes.

Our time‐resolved ChIP‐seq and transcriptomic analyses further show that LUH tunes the activities of distinct gene sets at specific stages. For instance, LUH binds *FYF*, a MADS‐box repressor of floral senescence and abscission (Chen et al., 2011), *AGL6*, a floral regulator contributing to meristem identity and floral organ specification (Yoo et al., 2011), *TEM2*, a repressor of FT and integrator of photoperiod and GA pathways (Castillejo & Pelaz, 2008; Matías‐Hernández et al., 2014), and *REM1*, implicated in reproductive meristem function (Franco‐Zorrilla et al., 2002). Binding at these loci at 2 and 8 DAI, but not at 4 DAI, is consistent with LUH acting on regulators of floral identity and phase transitions outside the peak of organ proliferation.

The *lug* FIS mutant exhibits premature floral organ differentiation due to misregulation of key floral identity and meristem maintenance genes, including upregulation of *AG* and *CRC* (Bowman & Smyth, 1999; Liu & Meyerowitz, 1995) and downregulation of *AP1* and *TFL1* (Ferrándiz et al., 2000). Comparison of transcriptional profiles from *lug* FIS and *luh* FIS mutants revealed a set of commonly misregulated genes that are also LUH‐bound, many of which are involved in developmental patterning and polarity. Consistently, LUH complexes were enriched for factors involved in regionalization, such as AINTEGUMENTA‐like 6 (AIL6) and KNOTTED‐LIKE FROM ARABIDOPSIS THALIANA (KNAT1) (Douglas et al., 2002; Krizek, 2011; Nole‐Wilson et al., 2005). These findings support a role for LUH, together with LUG and YABBY TFs, in establishing polarity axes during floral organogenesis (Navarro et al., 2004; Stahle et al., 2009). Importantly, our comparison reveals that LUG and LUH act on a shared target gene set with asymmetric contributions: LUG provides the predominant repression, whereas LUH contributes a weaker baseline layer. This functional asymmetry not only aligns with the partial redundancy model proposed previously (Sitaraman et al., 2008), but also extends it by showing that LUH recruitment is dynamically regulated across developmental stages. Specifically, our ChIP‐seq analyses indicate strong LUH recruitment during early patterning (2 DAI) and late consolidation/differentiation (8 DAI), with transient reconfiguration at peak organ outgrowth (4 DAI). Thus, LUH emerges not merely as a weaker partner of LUG but as a co‐regulator with distinct temporal dynamics, contributing an additional layer of complexity to co‐regulator‐mediated control of floral organogenesis.

In summary, LUH functions as a dynamic, stage‐specific co‐regulator that integrates developmental and environmental signals through interactions with a wide network of transcriptional and chromatin‐associated proteins. The transition in the protein complex composition of LUH and DNA‐binding behavior from meristematic to organogenic phases underscores a switch in its molecular activities in floral development. The extensive and evolving interaction network of LUH, overlapping yet distinct from LUG, highlights its function as an important coordinator of transcriptional regulation during early flower formation. The identification of LUH‐specific targets and co‐regulators expands our understanding of the gene regulatory logic underpinning floral morphogenesis.

## MATERIALS AND METHODS

### Plant material and growth conditions

To construct *proLUH:LUH‐GFP* in FIS, *proAP1:AP1‐GR ap1 cal* plants were transformed with *proLUH:LUH‐GFP* in the pGBGWG plasmid (Smaczniak, Immink, et al., 2012) following the floral dip method (Clough & Bent, 1998). For *proLUG:LUG(CDS)‐GFP* in FIS, the *LUG* coding sequence was amplified from a Col‐0 inflorescence cDNA pool and cloned into pCR8‐GW‐TOPO (Invitrogen) by TA cloning (for primer sequences see Table S6). An LR reaction was then performed to transfer *LUG(CDS)* into pMDC107 (Curtis & Grossniklaus, 2003). The *LUG* promoter (approximately 3.5 kb) was amplified from Col‐0 genomic DNA with SalI and ApaI restriction sites and inserted upstream of *LUG(CDS)* in *LUG(CDS):pMDC107*. The resulting *proLUG:LUG(CDS)‐GFP* construct was transformed into *proAP1:AP1‐GR ap1 cal* plants by floral dip (Clough & Bent, 1998). To construct *proSEU:SEU‐GFP* in FIS, *proSEU:SEU‐GFP* (Azhakanandam et al., 2008) plants were crossed with *proAP1:AP1‐GR ap1 cal* plants. The *proSEU:SEU‐GFP seu‐1* plants used for ChIP have been previously reported (Azhakanandam et al., 2008). To construct *lug* and *luh* mutants in FIS, the mutants *luh‐3* (Sitaraman et al., 2008) and *lug‐1* (Liu & Meyerowitz, 1995) were crossed with *proAP1:AP1‐GR ap1 cal* plants. Seeds were sterilized for 10 min with 1 ml of 5% sodium hypochlorite, 0.0025% Tween‐20 and then stratified in water for 2 days at 4°C in the dark. Seeds were sown on soil or on ½ MS plates and grown at 22°C with 16/8 h light (150 μmol m^−2^ sec^−1^)/dark cycles.

### Mucilage thickness estimation

To assess the biological activity of the *proLUH:LUH‐GFP* construct, the extent of mucilage release was assessed from seeds derived from *luh‐5/mum1‐1* (Western et al., 2001) mutant plants transformed by floral dipping (Clough & Bent, 1998). Approximately 50 seeds from 16 independent lines, as well as from wildtype and *luh‐5* plants grown alongside the T1 lines were agitated in distilled water for 90 min, before staining for 30 min in freshly prepared 0.02% (w/v) Ruthenium Red (RR) solution. Stained seeds were rinsed twice in water before imaging under a dissecting microscope. Using ImageJ, two measurements of adherent mucilage thickness were obtained from opposite sides of over 35 seeds at a position halfway along the length of the seed and subsequently averaged.

### Flowering induction of the FIS plants

The inflorescences of FIS plants were treated with 2 μm dexamethasone, 0.0001% (v/v) DMSO, and 0.015% (v/v) Silwet L‐77. For mock treatments, dexamethasone was omitted. Plants were induced daily at 10 a.m. in 24‐h intervals and the tissue was collected at 4 p.m. on a specific DAI.

### Phenotypic analysis of inflorescences

Inflorescences of *luh* FIS, *lug* FIS, and FIS plants at specific DAI were collected and directly mounted on a charcoal‐agar solid plate. The photos were taken with a DVM6 digital microscope (Leica Microsystems, Wetzlar, Germany).

### Confocal microscopy

The tip of the inflorescence at a specific DAI was dissected and embedded in a solution of 0.15% agarose in a custom chamber on a microscope slide. All the images were taken with the LSM 800 upright confocal microscope (Zeiss, Oberkochen, Germany) equipped with 10× and 20× Plan‐Apochromat as well as 40× and 63× C‐Apochromat objectives. GFP was excited with a 488‐nm argon laser and the GFP emission signal was recorded with a 410–540 nm bandpass filter on the GaAsP‐Pmt1 detector. Chlorophyll autofluorescence from chloroplasts was recorded with a 650–700 nm bandpass filter on the GaAsP‐Pmt2 detector. Z‐stack images were merged into 3D projections in the ZEN blue v2.3 imaging software (Zeiss). Airyscan images were taken with the 63× C‐Apochromat objective with the Airyscan detector. To stain plant cell walls, tissues were incubated in 1 mg ml^−1^ propidium iodide for 2 min.

### *In vitro* co‐immunoprecipitation (Co‐IP)

Coding sequences of the proteins used in Co‐IPs were cloned from the pool of cDNA generated from the Col‐0 wild‐type inflorescence material (for primer sequences, see Table S6) and inserted in the pSPUTK *in vitro* translation vector (Stratagene, La Jolla, CA, USA). The *in vitro* protein synthesis was done using SP6 High‐Yield Wheat Germ Protein Expression System (Promega, Madison, WI, USA) in the presence of Transcend (biotinylated lysine)‐tRNA (Promega) in 10 μl reactions, following the manufacturer's instructions; for Co‐IP‐MS, biotinylation was omitted. Proteins were co‐synthesized in a single tube from equimolar plasmid concentrations. One fifth of each reaction was denatured in Elution Buffer (Miltenyi Biotec, Bergisch Gladbach, Germany) and used as an input control. The remaining proteins were diluted with Lysis Buffer (Miltenyi Biotec) and precipitated with AP1 or SEP3 antibodies (custom rabbit polyclonal; Eurogentec, Seraing, Belgium) by mixing at 4°C for 1 h. In these assays, AP1 and SEP3 served as bait proteins for immunoprecipitation. Immunoprecipitated protein complexes were captured with μMACS Protein A MicroBeads (Miltenyi Biotec) mixing at 4°C for 1 h, passed through a magnetic μColumn (Miltenyi Biotec) fixed on a μMACS Separator (Miltenyi Biotec), and washed four times with Wash Buffer 1 and one time with Wash Buffer 2 (Miltenyi Biotec). Proteins were eluted either with hot Elution Buffer (Miltenyi Biotec) for Western blot or with 8 m urea for subsequent LC–MS/MS analysis. Western blot signals were detected using Streptavidin–Alkaline Phosphatase (Promega) and Western Blue Substrate (Promega) since proteins were synthesized with biotinylated lysine residues, enabling visualization of both the immunoprecipitated proteins (AP1 or SEP3) and their interactors (LUH or SEU) based on migration in the gel. LC–MS/MS analysis was performed as described below.

### Immunoprecipitation followed by mass spectrometry (IP‐MS)

Immunoprecipitation followed by mass spectrometry was performed essentially as described before (Smaczniak, Li, et al., 2012) using an updated protocol (Smaczniak, 2023). Three biological replicates were processed. In brief, approximately 0.8 g of inflorescences were collected and ground in liquid nitrogen followed by nuclei isolation and nuclear protein extraction. GFP‐tagged proteins were immunoprecipitated with anti‐GFP micro beads (Miltenyi Biotec) mixing at 4°C for 1 h. The samples were passed through a magnetic μColumn (Miltenyi Biotec) fixed on a μMACS Separator (Miltenyi Biotec) and washed six times with Lysis Buffer and two times with Wash Buffer 2 (Miltenyi Biotec). The immunoprecipitates were eluted from the column with 8 m urea.

The IP samples were digested in solution with trypsin while the corresponding input samples (nuclear protein extracts) were digested following the FASP protocol (Wiśniewski et al., 2009). All peptide samples were desalted and run on an HPLC column using the EASY‐nLC 1200 System (Thermo Fisher Scientific, Waltham, MA, USA) online with the Q Exactive Plus mass spectrometer (Thermo Fisher Scientific). The output files were processed with MaxQuant (Cox & Mann, 2008) and statistical analysis was done with Perseus (Tyanova et al., 2016). Proteins initially assigned to “protein groups” in MaxQuant were reported individually to enable downstream network analyses; however, we note that these proteins cannot be distinguished unambiguously based on the available peptide evidence. The graphs were plotted with R.

### Chromatin immunoprecipitation followed by sequencing (ChIP‐seq)

ChIP‐seq was performed essentially as described before (Kaufmann et al., 2010; van Mourik et al., 2015) with several modifications. Two biological replicates were processed for each sample, except for the LUH‐GFP 8 DAI sample, for which three biological replicates were available. Approximately 1 g of inflorescences were collected and incubated with 2.5 mm DSG and 0.01% (v/v) DMSO for the primary fixation. The samples were placed under vacuum for 20 min in a Concentrator plus (Eppendorf, Hamburg, Germany). For the secondary fixation, formaldehyde was added to 1% (v/v) concentration, samples were mixed and placed under vacuum for 20 min. The crosslinking reaction was stopped by adding glycine. The fixed tissue was ground in liquid nitrogen followed by nuclei isolation and chromatin extraction. The chromatin was sonicated using the S220 (Covaris, Woburn, MA, USA) with the following settings: Peak Power = 140.0; Cycles/Burst = 200; Duty Factor = 5.0; Duration = 540 sec; temp = 6°C. Samples were centrifuged and a portion of the sonicated chromatin was set aside as input control. For LUH‐GFP ChIP (*proLUH:LUH‐GFP* in FIS), GFP antibodies (ab290, Abcam, Cambridge, UK) were added and mixed at 4°C for 1 h. The immunoprecipitated chromatin was captured with μMACS Protein A MicroBeads (Miltenyi Biotec) mixing at 4°C for 1 h. For SEU‐GFP ChIP (*proSEU:SEU‐GFP seu‐1*), μMACS Anti‐GFP MicroBeads (Miltenyi Biotec) were added and mixed at 4°C for 1 h. The samples were passed through a magnetic μColumn (Miltenyi Biotec) fixed on a μMACS Separator (Miltenyi Biotec) and washed two times with IP buffer, two times with high‐salt buffer, two times with LiCl buffer, and two times with 1× TE buffer. The immunoprecipitated chromatin was eluted with hot elution buffer. To reverse the crosslinking, input and eluate samples were incubated with 250 mm NaCl in a thermomixer overnight at 65°C and 1000 rpm, followed by 1 h incubation with 0.615 mg ml^−1^ proteinase K at 65°C and 1000 rpm. The de‐crosslinked DNA was ethanol‐precipitated and purified using the DNA Clean and Concentrator kit (Zymo Research, Irvine, CA, USA).

ChIP‐seq library was prepared with the ThruPLEX DNA‐seq Kit (Takara Bio, Shiga, Japan) following the manufacturer's instructions. The libraries were loaded on a 1% low melting point agarose gel, and 100–700 bp DNA fragments were purified with the MiniElute Gel Extraction kit (Qiagen, Hilden, Germany). The purified libraries were assessed on a 4200 TapeStation (Agilent Technologies, Santa Clara, CA, USA) and the concentration was measured using the Qubit 4 (Invitrogen, Waltham, MA, USA). The libraries were sequenced using Illumina (San Diego, CA, USA) high‐throughput sequencing devices.

### ChIP‐seq data analysis

The quality of the raw sequencing reads was assessed with *FastQC*. Potential adapter sequences were removed from the sequencing reads using *Trimmomatic* (Bolger et al., 2014) and the quality of trimmed reads was reassessed. Trimmed reads were mapped to the Arabidopsis TAIR10 reference genome using *Bowtie2* (Langmead & Salzberg, 2012). Only reads with MAPQ score >40 were kept. Peak calling and fragment pileup was performed using *MACS2* (Zhang et al., 2008). For LUH‐GFP ChIP‐seq, the regions declared significant by *MACS2* were merged using *mergeBED* from the *bedtools* package (Quinlan & Hall, 2010) with parameters: *‐iobuf 5G ‐d 100* to obtain a common reference set to later test with *DESeq2* (Love et al., 2014). For SEU‐GFP ChIP‐seq, *MACS2* peak calls from two biological replicates were processed with irreproducibility discovery rate (IDR) (Li et al., 2011) with a threshold set to 0.05. The peak annotation was done considering the closest TSS of a gene in a range of 3 kb from the center of the peak. ChIP‐seq fragment pileups were visualized with the *IGV Web App* (Robinson et al., 2011). The called peaks were further evaluated with *ChIPseeker* (Yu et al., 2015). Significantly called peaks were compared to other ChIP‐seq experiments collected in the ChIP‐Hub (Fu et al., 2022) using the Jaccard similarity index using *jaccard* tool from the *bedtools* package (Quinlan & Hall, 2010) using default parameters.

### Transcriptomic profiling by RNA‐seq

Three biological replicates for each condition were processed. Inflorescences were collected and immediately frozen in liquid nitrogen. The tissue was homogenized with the Precellys 24 (Bertin Technologies, Montigny‐le‐Bretonneux, France) homogenizer and the RNA was isolated with the QIAwave RNA Mini Kit (Qiagen). DNA was removed by DNase I digestion followed by LiCl precipitation. The RNA concentration was measured using the Qubit 4 (Invitrogen) and the RNA integrity was assessed on a 4200 TapeStation (Agilent Technologies). Samples with an RNA integrity number equivalent (RINe) higher than 6 were considered for further processing. Approximately 300 ng of total RNA served as input for library preparation using the TruSeq Stranded mRNA LT Sample Prep Kit (Illumina). Libraries were run on the 4200 TapeStation (Agilent Technologies) for validating the quality, the correct DNA fragment size, and the absence of adaptor contamination.

### RNA‐seq data analysis

The raw sequencing reads were trimmed using *Trimmomatic* (Bolger et al., 2014). The trimmed reads were mapped to the Arabidopsis TAIR10 genome/transcriptome using *STAR* (Dobin et al., 2013) with parameters: *‐‐alignIntronMax 10 000 ‐‐outFilterMultimapNmax 1 ‐‐outSJfilterReads Unique*. The software *featureCounts* (Liao et al., 2014) was used to count the number of reads per gene, using the parameters: *‐t gene ‐g gene_id ‐T 20 ‐p*. Only genes with at least 10 read counts in at least two RNA‐seq samples were considered. Genes encoded in the chloroplast or mitochondria genomes were eliminated. *DESeq2* (Love et al., 2014) was used to normalize read counts across samples and to identify DEGs. DEGs were defined as those with an adjusted *P*‐value (FDR) <0.05 and |log_2_FC| > 1.

### Other computational analyses

Heatmaps were plotted in R using the *pheatmap* package. Venn diagrams were plotted in R using *VennDiagram* package. All Gene Ontology analyses were performed using the ShinyGO platform (Ge et al., 2020). The protein–protein interaction networks were built using the STRING platform (Szklarczyk et al., 2023). Significant interactors with at least 16 times enrichment (*P* < 0.05, log_2_FC > 4) from each stage were uploaded. This stringent cutoff was used to define high‐confidence interactors for protein network plotting. A physical subnetwork with a medium confidence value of 0.400, using all the interaction sources, and a maximum of 10 interactors to show in the first shell was set. The enriched motifs were identified using the MEME‐ChIP online platform (Machanick & Bailey, 2011). To avoid a bias from repetitive sequences, all the FASTA files used in the analysis were masked. DAP motifs (O'Malley et al., 2016) were set as the reference database.

## AUTHOR CONTRIBUTIONS

RV‐L, KK, and CS conceived and designed the research; RV‐L performed most of the experiments with contributions from JAG, JFG, MP, KR, SS, ER, and CS; RV‐L, and JMM analyzed most of the data with contributions from TZ, DC and CS; RV‐L, KK, and CS wrote the paper.

## CONFLICT OF INTEREST

The authors declare no conflict of interest.

## Supporting information


**Figure S1.** Expression pattern of *proLUH:LUH‐GFP* in Col‐0 background at different stages of flower development.
**Figure S2.** Expression pattern of *proLUG:LUG(CDS)‐GFP* in FIS and *proSEU:SEU‐GFP* in FIS.
**Figure S3.** Dynamic changes of LUH protein interaction partners across different stages of flower development in the IP‐MS eluates.
**Figure S4.** Protein enrichment versus gene expression dynamics across different DAI samples for selected groups of proteins.
**Figure S5.** Co‐expression heatmap of the co‐regulator and histone modifier hub members with the TraVA DB expression database.
**Figure S6.** Expression of proteins used in Co‐IPs in the *in vitro* wheat germ extract system.
**Figure S7.** Unedited LUH Co‐IP Western blots used in Figure 4.
**Figure S8.** Unedited SEU Co‐IP Western blots used in Figure 4.
**Figure S9.** LUH occupancy characteristics during different stages of flower development.
**Figure S10.** Phenotypic analysis of FIS, *luh* FIS, and *lug* FIS inflorescences at different stages of flower development.
**Figure S11.** Reproducibility of relative expression values from independent biological replicates of FIS, *luh* FIS, and *lug* FIS inflorescences.
**Figure S12.** Gene expression analysis (*luh* FIS versus FIS) of co‐bound genes in LUH and other TF ChIP‐seq across different stages of flower development.
**Table S1.** Complementation of the *luh‐5* seed mucilage defect by *proLUH:LUH‐GFP*.
**Table S2.** Co‐IP‐MS proteomics data for LUH and AP1 proteins.
**Table S3.** Number of significant peaks and their closest associated genes identified in ChIP‐seq experiments.
**Table S4.** Number of differentially expressed genes identified in RNA‐seq experiments.
**Table S5.** Jaccard similarity indices for pairwise ChIP‐seq experiment comparisons.
**Table S6.** Primers used for cloning LUH and SEU constructs.

**Data S1.** Protein enrichment analysis of the LUH‐GFP IP‐MS samples at different stages of flower development.

**Data S2.** Gene Ontology enrichment analysis of significantly enriched proteins in the LUH‐GFP IP‐MS samples at different stages of flower development.

**Data S3.** Peak calling results including gene annotation for the LUH‐GFP ChIP‐seq samples at different stages of flower development.

**Data S4.** Gene Ontology enrichment analysis of the LUH‐GFP ChIP‐seq target genes at different stages of flower development.

**Data S5.** Raw Jaccard similarity index values calculated between LUH ChIP‐seq datasets and datasets from ChIP‐hub.

**Data S6.** SEU‐GFP (seu‐1) ChIP‐seq datasets, including peak calls, GO enrichment, and overlap with LUH targets.

**Data S7.** Integrated analysis of LUH ChIP‐seq and RNA‐seq, including GO annotation of differentially expressed LUH targets.

**Data S8.** Differentially expressed genes in *luh* FIS, *lug* FIS and FIS RNA‐seq samples at different stages of flower development.

**Data S9.** Differentially expressed genes in the *luh FIS* mutant among LUH‐TF and LUH‐cofactor co‐bound targets.

## Data Availability

The mass spectrometry proteomics data have been deposited to the ProteomeXchange Consortium via the PRIDE (Perez‐Riverol et al., 2025) partner repository with the dataset identifiers PXD060520 and PXD068765. The ChIP‐seq and RNA‐seq data discussed in this publication have been deposited in NCBI's Gene Expression Omnibus (Edgar et al., 2002) and are accessible through GEO Series accession numbers GSE289372, GSE289681, and GSE294839.
