# Supplementary material for: Rewiring of LEUNIG_HOMOLOG interaction networks marks regulatory shifts from meristem to organ growth in Arabidopsis flowers
Source: Plant J. 2025 Nov 24;124(4):e70585. doi: 10.1111/tpj.70585 (PMC12643531; doi:10.1111/tpj.70585)
Supplement: Supplementary file 1 — Figure S1. Expression pattern of proLUH:LUH‐GFP in Col‐0 background at different stages of flower development. Figure S2. Expression pattern of proLUG:LUG(CDS)‐GFP in FIS and proSEU:SEU‐GFP in FIS. Figure S3. Dynamic changes of LUH protein interaction partners across different stages of flower development in the IP‐MS eluates. Figure S4. Protein enrichment versus gene expression dynamics across different DAI samples for selected groups of proteins. Figure S5. Co‐expression heatmap of the co‐regulator and histone modifier hub members with the TraVA DB expression database. Figure S6. Expression of proteins used in Co‐IPs in the in vitro wheat germ extract system. Figure S7. Unedited LUH Co‐IP Western blots used in Figure 4. Figure S8. Unedited SEU Co‐IP Western blots used in Figure 4. Figure S9. LUH occupancy characteristics during different stages of flower development. Figure S10. Phenotypic analysis of FIS, luh FIS, and lug FIS inflorescences at different stages of flower development. Figure S11. Reproducibility of relative expression values from independent biological replicates of FIS, luh FIS, and lug FIS inflorescences. Figure S12. Gene expression analysis (luh FIS versus FIS) of co‐bound genes in LUH and other TF ChIP‐seq across different stages of flower development. Table S1. Complementation of the luh‐5 seed mucilage defect by proLUH:LUH‐GFP. Table S2. Co‐IP‐MS proteomics data for LUH and AP1 proteins. Table S3. Number of significant peaks and their closest associated genes identified in ChIP‐seq experiments. Table S4. Number of differentially expressed genes identified in RNA‐seq experiments. Table S5. Jaccard similarity indices for pairwise ChIP‐seq experiment comparisons. Table S6. Primers used for cloning LUH and SEU constructs. [file TPJ-124-0-s005.docx]

**Supporting Information**

**Supporting figures**


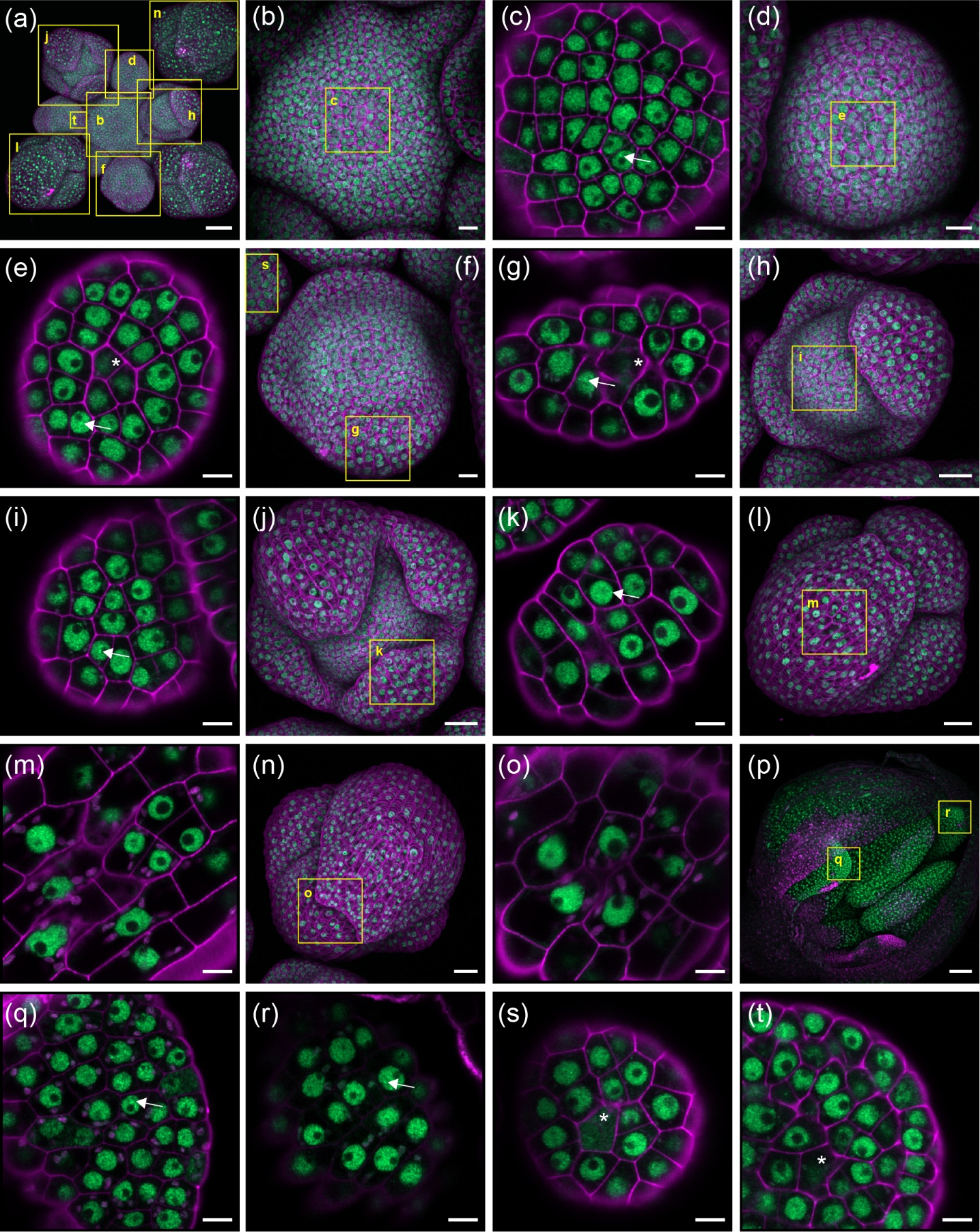


**Figure S1.** Expression pattern of *proLUH:LUH-GFP* in Col-0 background at different stages of flower development. **(A)** An overview confocal image of the plant inflorescence with flower buds at different developmental stages (stages 1-7). Yellow tiles indicate further images taken with higher magnifications. **(B, D, F, H, J, L, N)** Orthogonal projections of flower buds indicated in **(A)** taken with a higher magnification. Yellow tiles indicate regions of individual Z-stack images taken with the high-resolution Airyscan. **(C, E, G, I, K, M, O, Q-T)** Individual Z-stack Airyscan images from the corresponding images. Flower developmental stages: **(B-C)** inflorescence meristem; **(D-E)** stage 2; **(F-G)** stage 3; **(H-I)** stage 4; **(J-K)** stage 5; **(L-M)** stage 6; **(N-O)** stage 7; **(P-R)** stage 9; **(S)** stage 1-2; **(T)** stage 2-3. White arrows indicate nuclear condensates. Asterisks indicate diffused GFP signal of dividing cells. Magenta signal marks cell wall staining by propidium iodide. Scale bars are: 5 µm **(C, E, G, I, K, M, O, Q-T)**; 10 µm **(B, D, F)**; 20 µm **(H, J, L, N)**, 50 µm **(A, P)**.


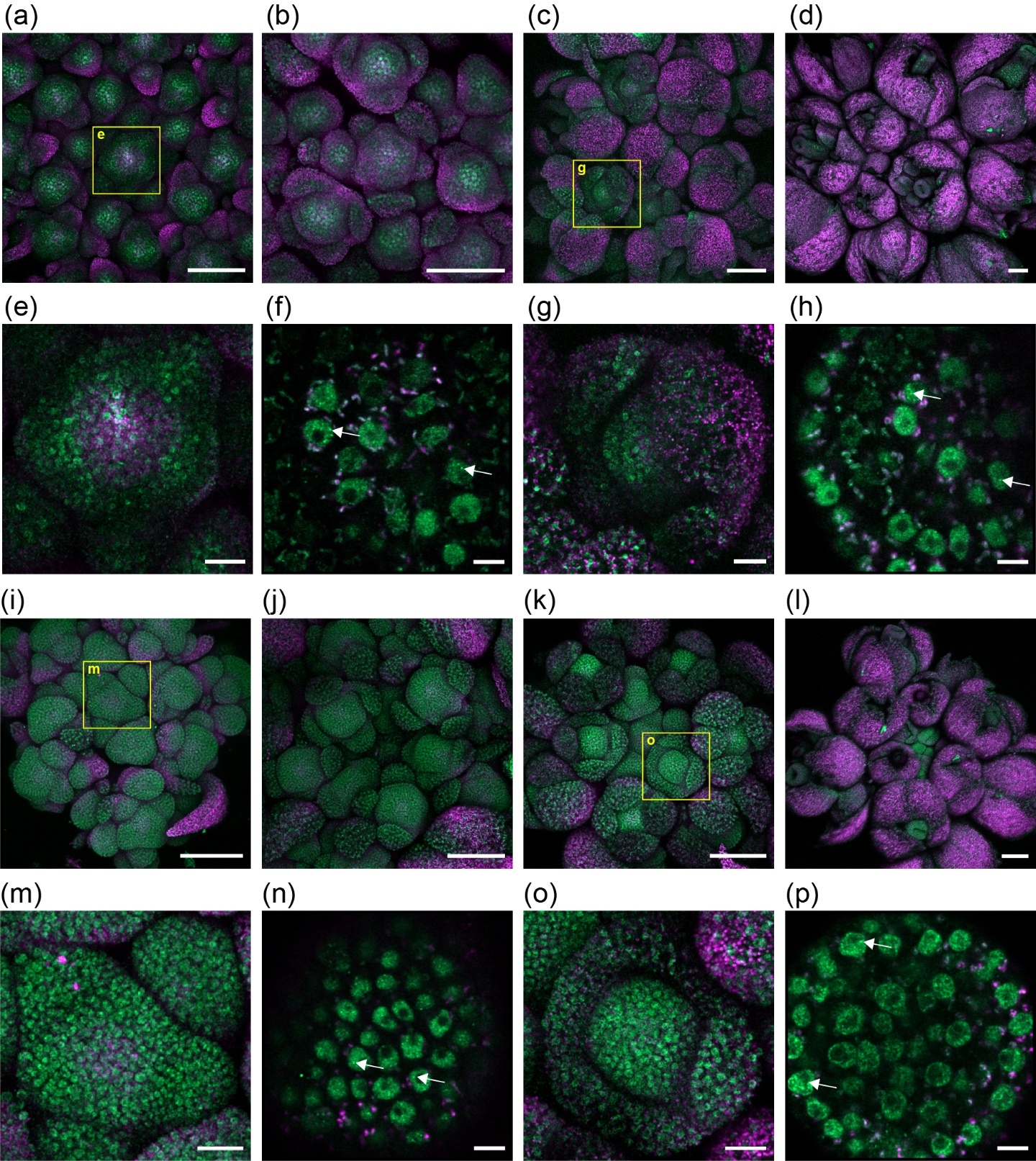


**Figure S2.** Expression pattern of *proLUG:LUG(CDS)-GFP* in FIS and *proSEU:SEU-GFP* in FIS. **(A-H)** *pLUG:LUG(CDS)-GFP* in FIS; **(I-P)** *proSEU:SEU-GFP* in FIS; **(A, E, F, I, M, N)** 0 DAI; **(B, J)** 2 DAI; **(C, G, H, K, O, P)** 4 DAI; **(D, L)** 8 DAI. Yellow outlines mark regions that were subsequently imaged at higher magnification. **(E, G, I, K)** are tissue regions from corresponding panels taken with higher magnifications. **(F, H, N, P)** show individual Z-stack images of tissues acquired with high-resolution Airyscan. Arrows exemplify nuclear condensates. Magenta signal marks chloroplast autofluorescence. Scale bars are 100 µm in **(A-H)** and 5 µm in **(I-J)**.


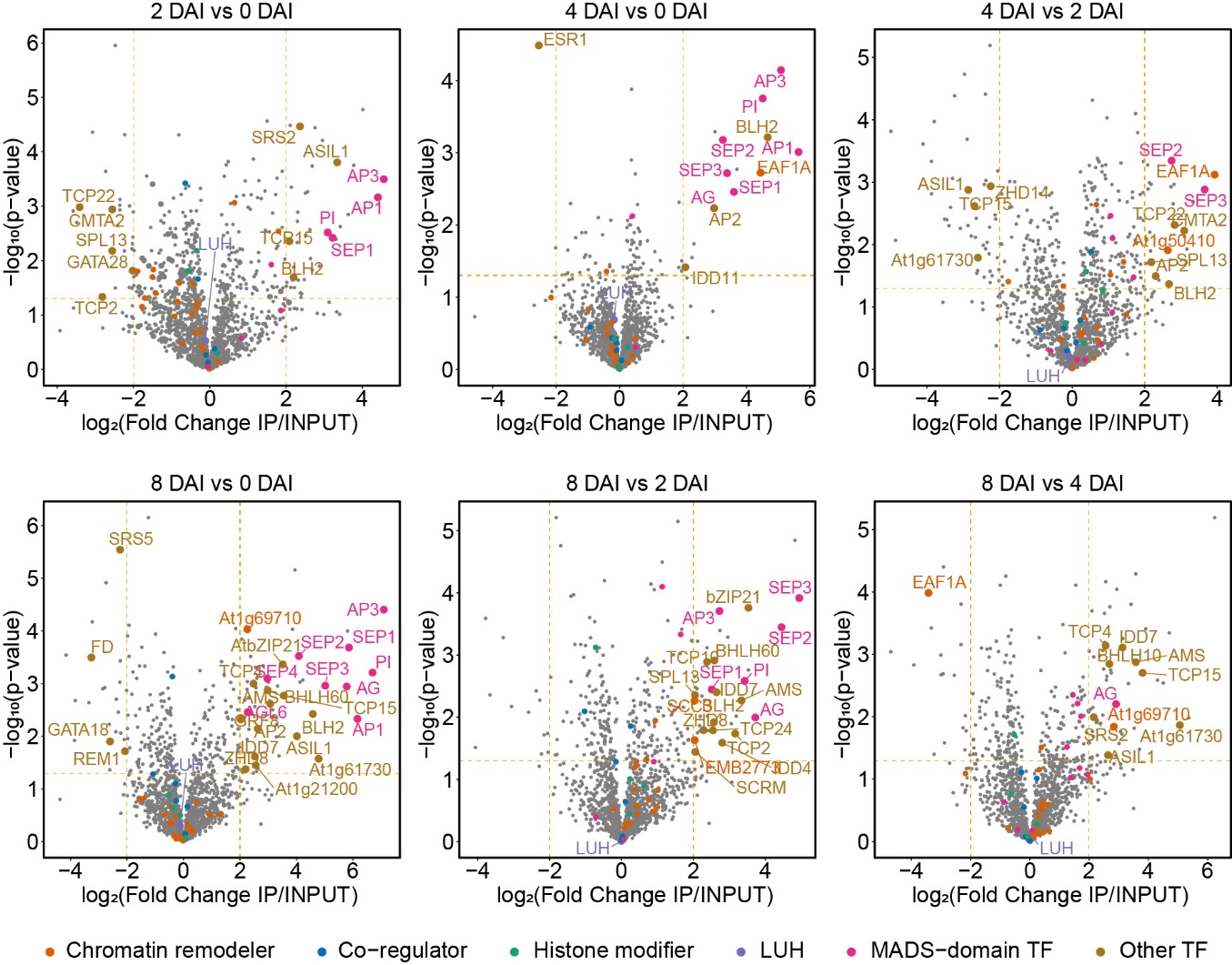


**Figure S3.** Dynamic changes of LUH protein interaction partners across different stages of flower development in the IP-MS eluates. Dashed lines mark thresholds: |log_2_*FC*| = 2 and *p*-value = 0.05. Only selected protein names are marked.


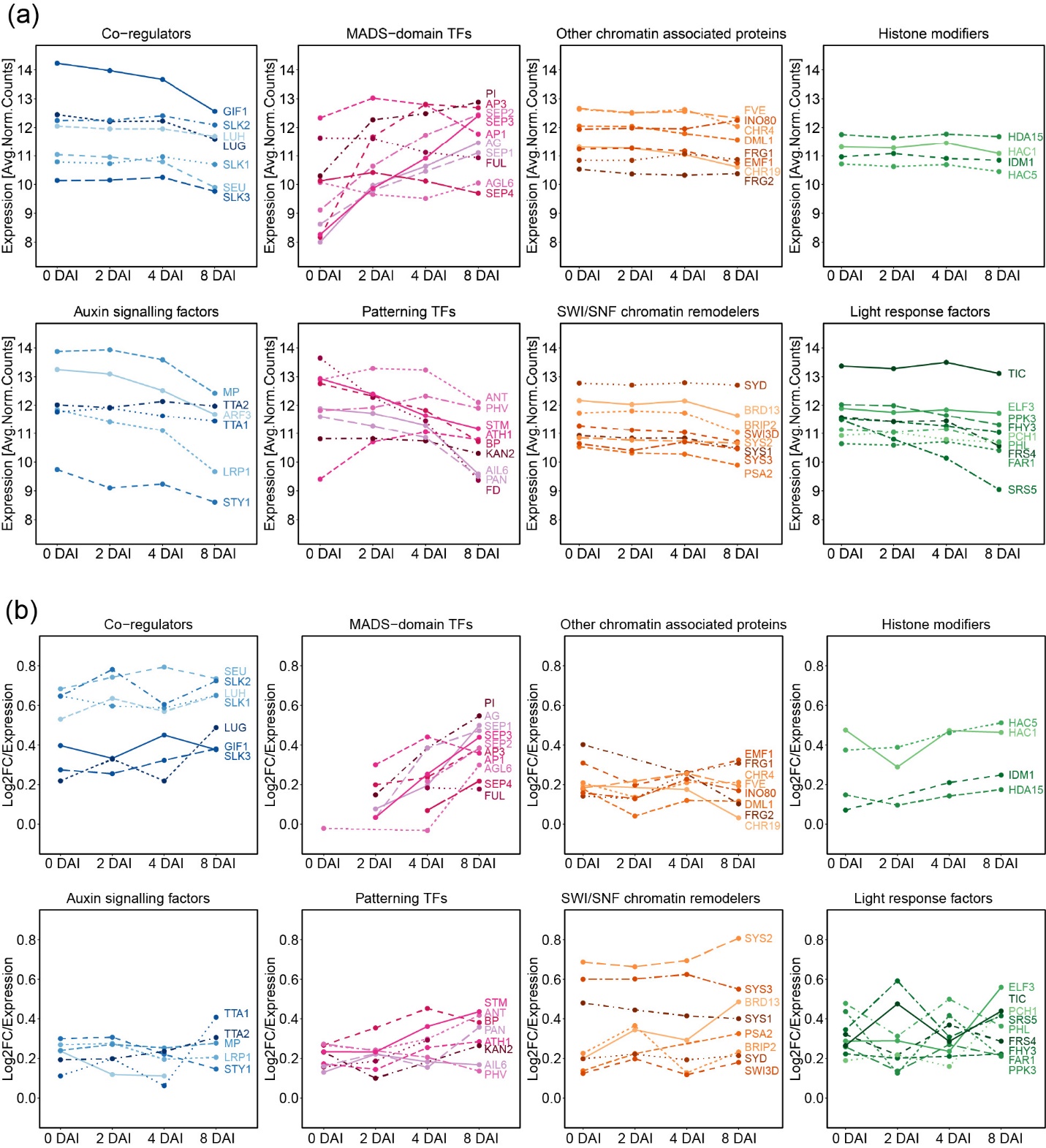


**Figure S4.** Protein enrichment vs. gene expression dynamics across different DAI samples for selected groups of proteins. **(A)** Normalized gene expression changes across different developmental time points. **(B)** Protein enrichment in the IP-MS experiments (log_2_*FC*) divided by the corresponding gene expression.


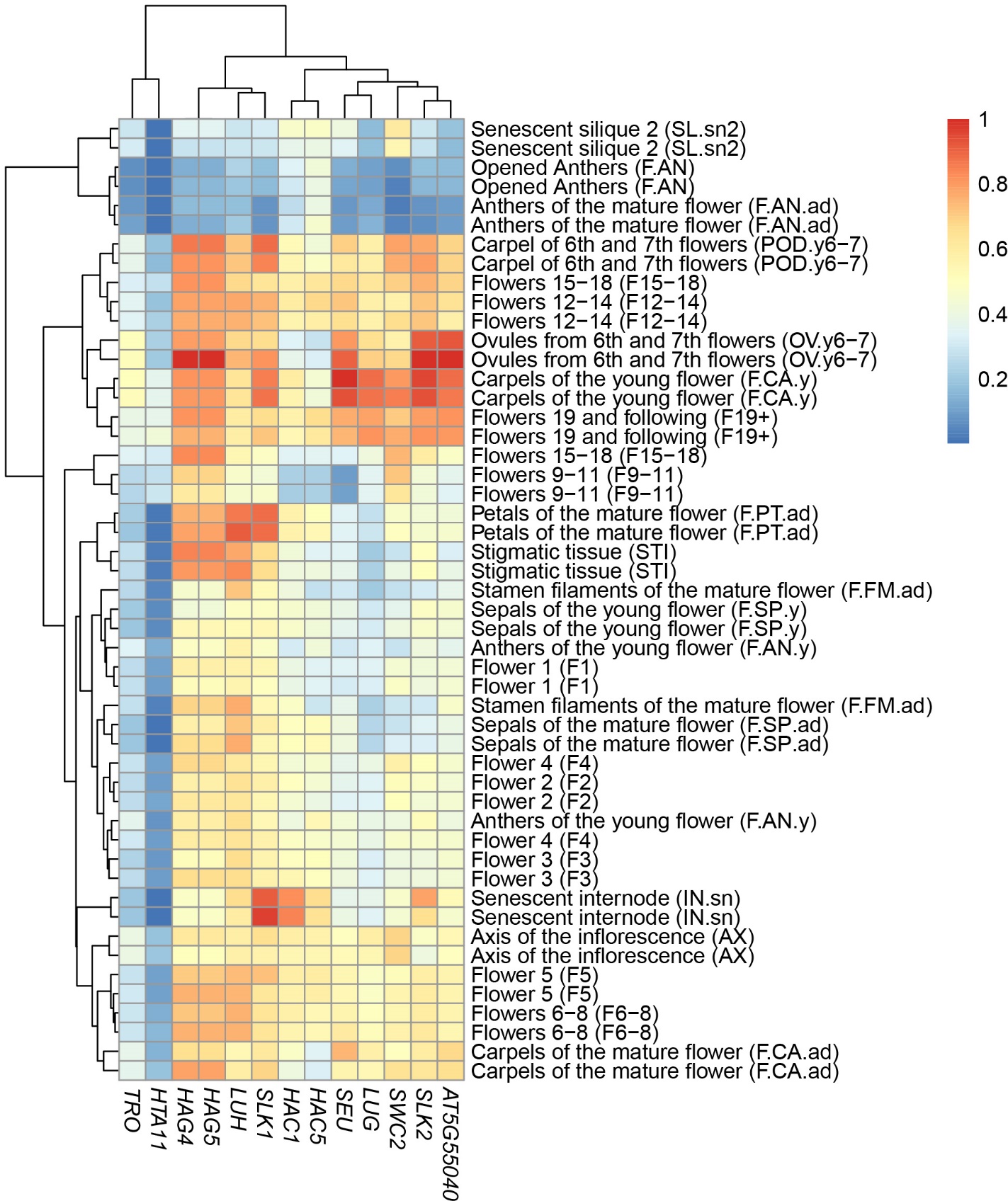


**Figure S5.** Co-expression heatmap of the co-regulator and histone modifier hub members with the TraVA DB expression database. The scale represents a normalized TPM (Transcripts Per Million). The names of tissues are TraVA DB-specific.


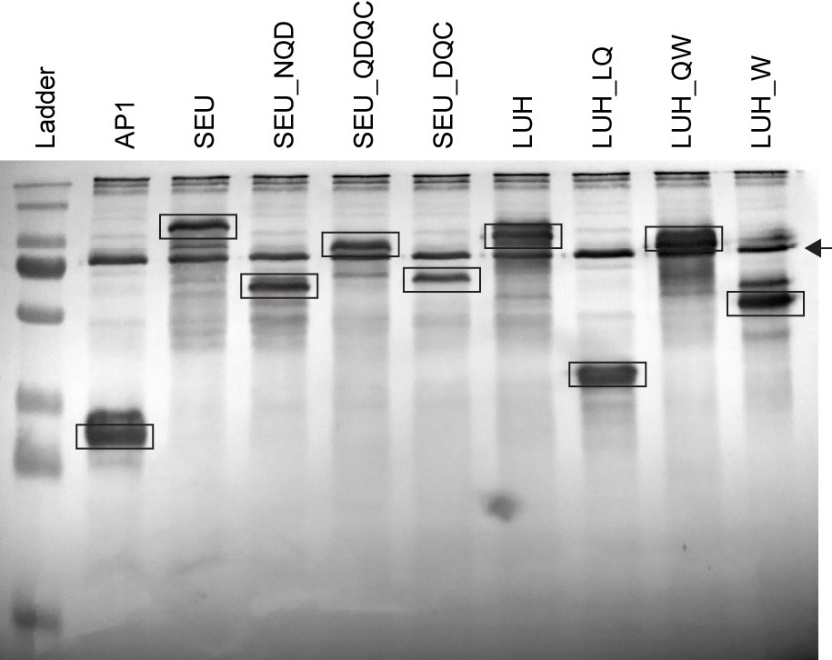


**Figure S6.** Expression of proteins used in Co-IPs in the *in vitro* wheat germ extract system. Boxes indicate the proteins of interest. The arrow indicates wheat germ expression system background signal.


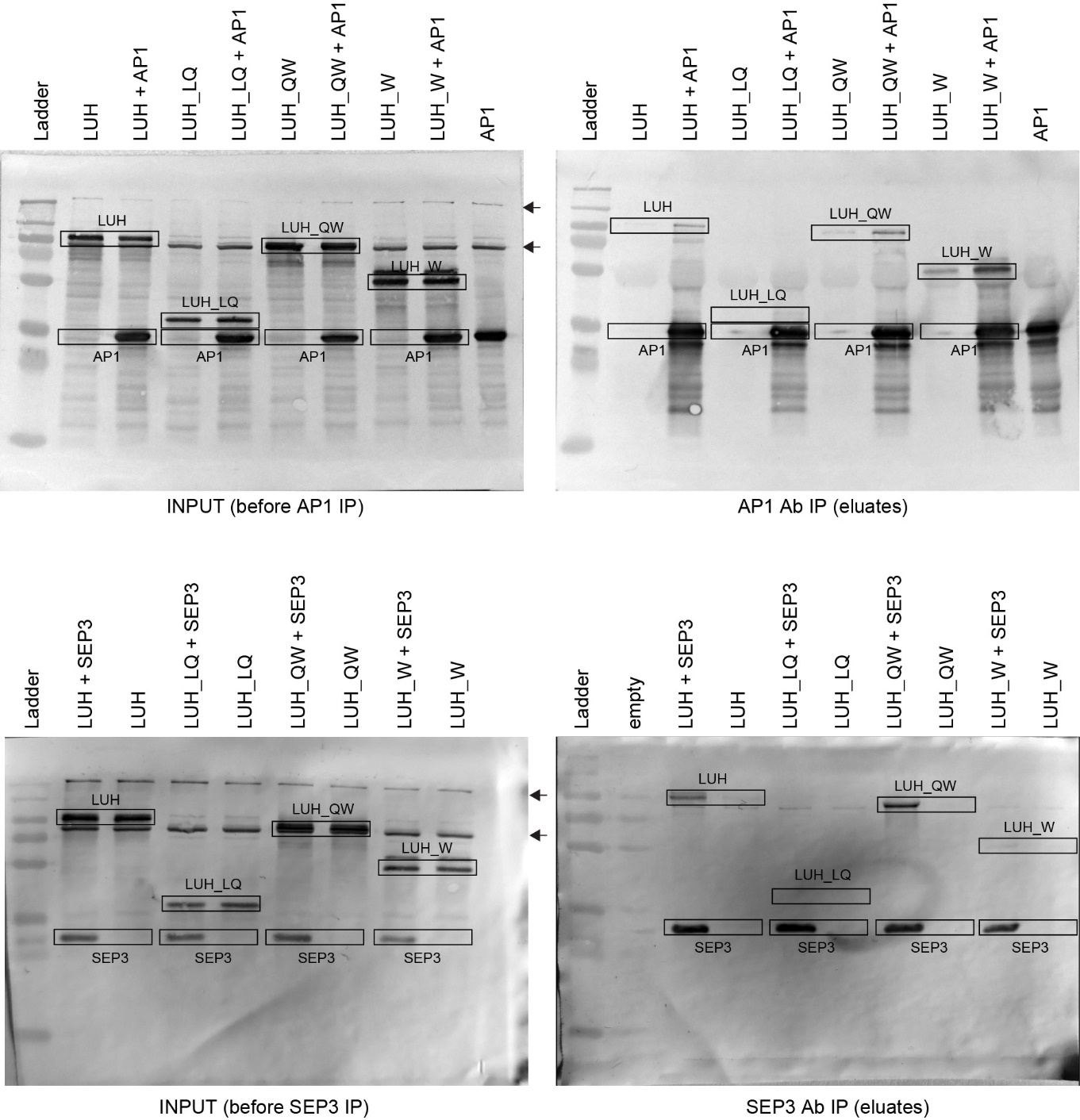


**Figure S7.** Unedited LUH Co-IP Western blots used in Figure 4. Arrows indicate wheat germ expression system background signal. Boxes highlight the regions of the blots that were used to assemble Figures 4b and 4c.


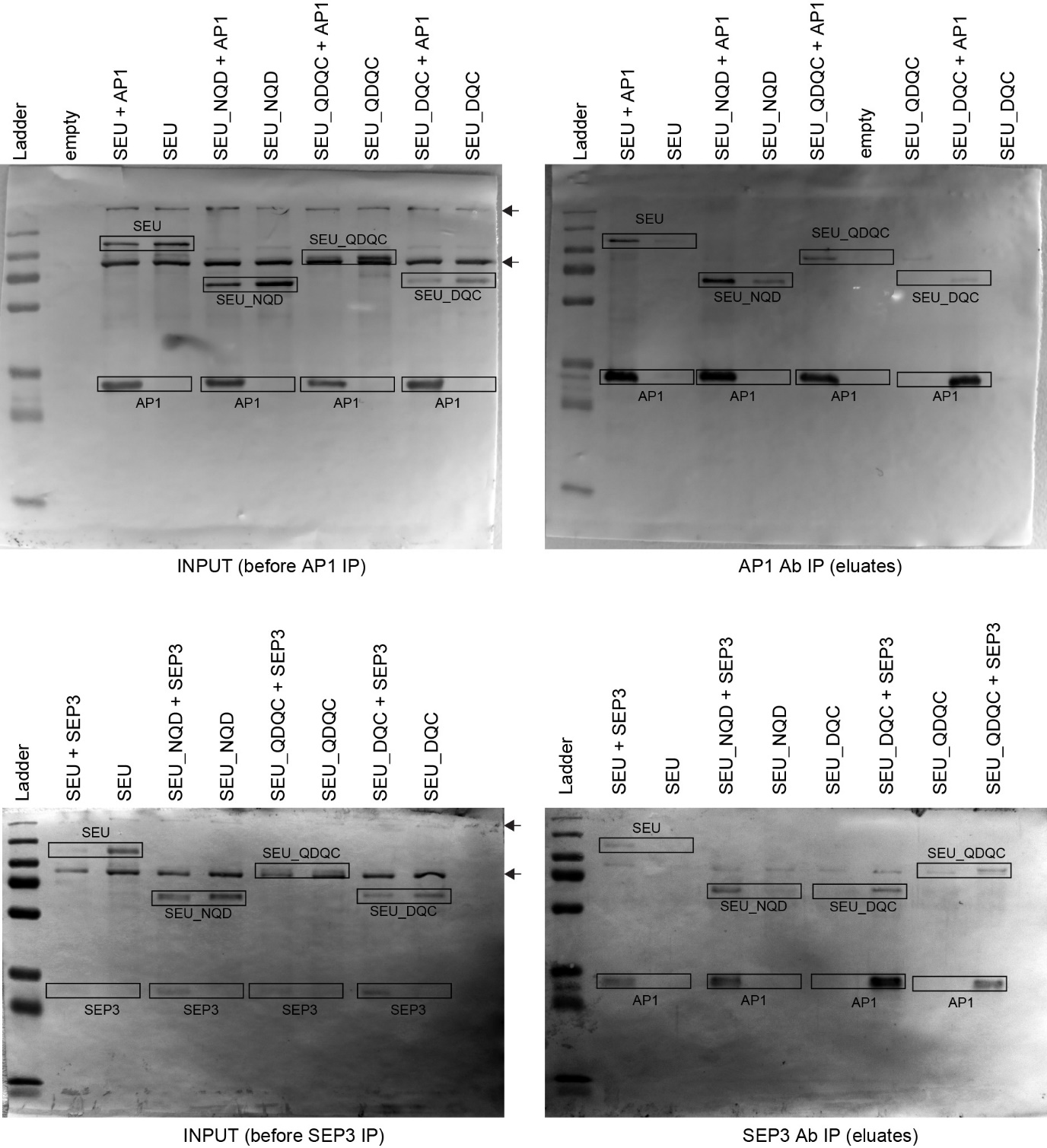


**Figure S8.** Unedited SEU Co-IP Western blots used in Figure 4. Arrows indicate wheat germ expression system background signal. Boxes highlight the regions of the blots that were used to assemble Figures 4f and 4g.


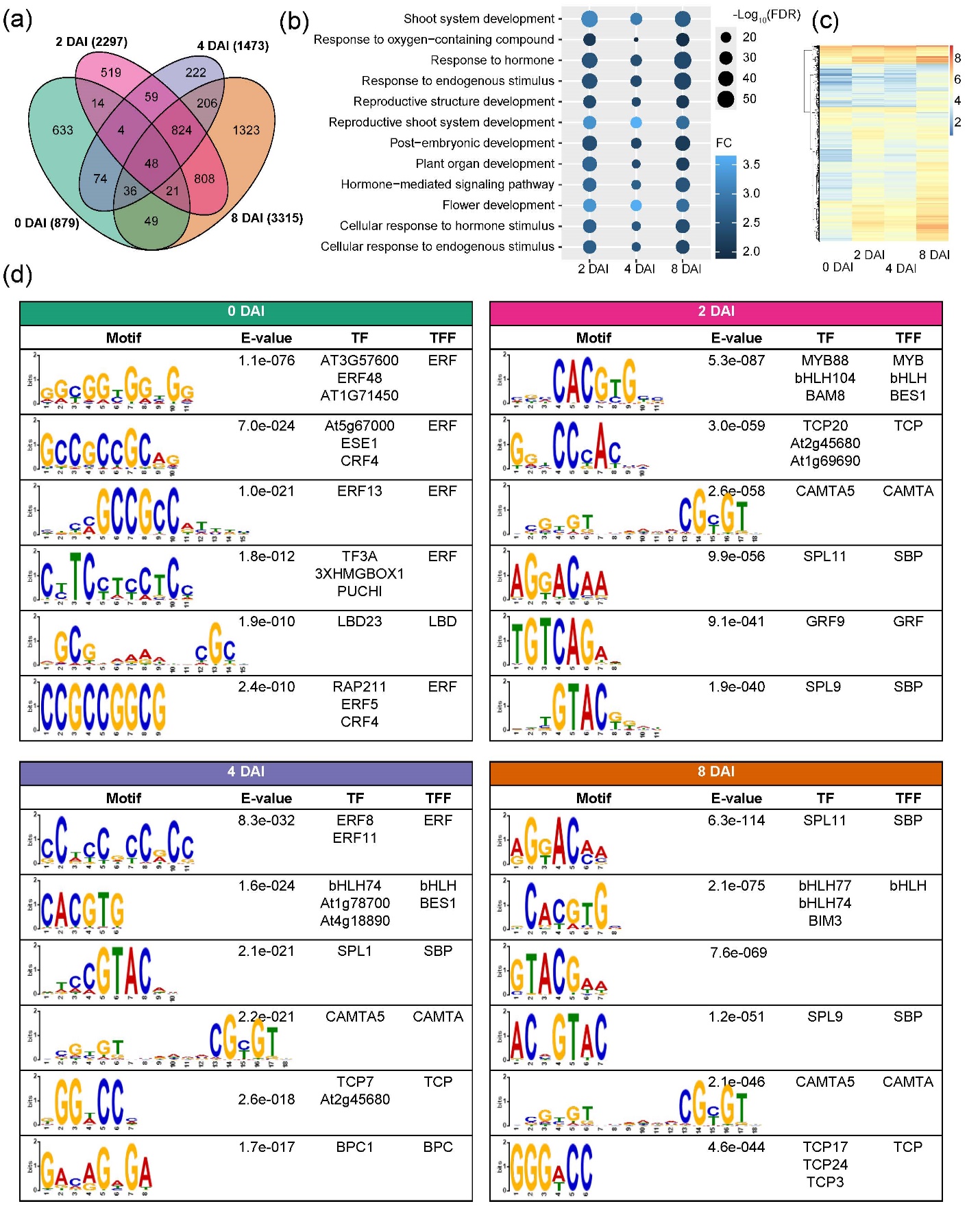


**Figure S9.** LUH occupancy characteristics during different stages of flower development. **(A)** Venn diagram of common and specific target genes of LUH. Only genes closest (3 kb distance) to a significant peak (*p*-value < 0.05) were considered. **(B)** GO enrichment for the LUH target genes with the GO BP categories. Sample 0 DAI is not shown because no GO BP enrichment was found at FDR < 0.001. **(C)** Heatmap of raw ChIP-seq peak intensities for LUH DNA-binding peaks at different stages of flower development. **(D)** Top 6 enriched motifs detected in ChIP-seq of LUH at each stage by MEME-ChIP suite. E-value after MEME-ChIP. TFF: Transcription factor family.


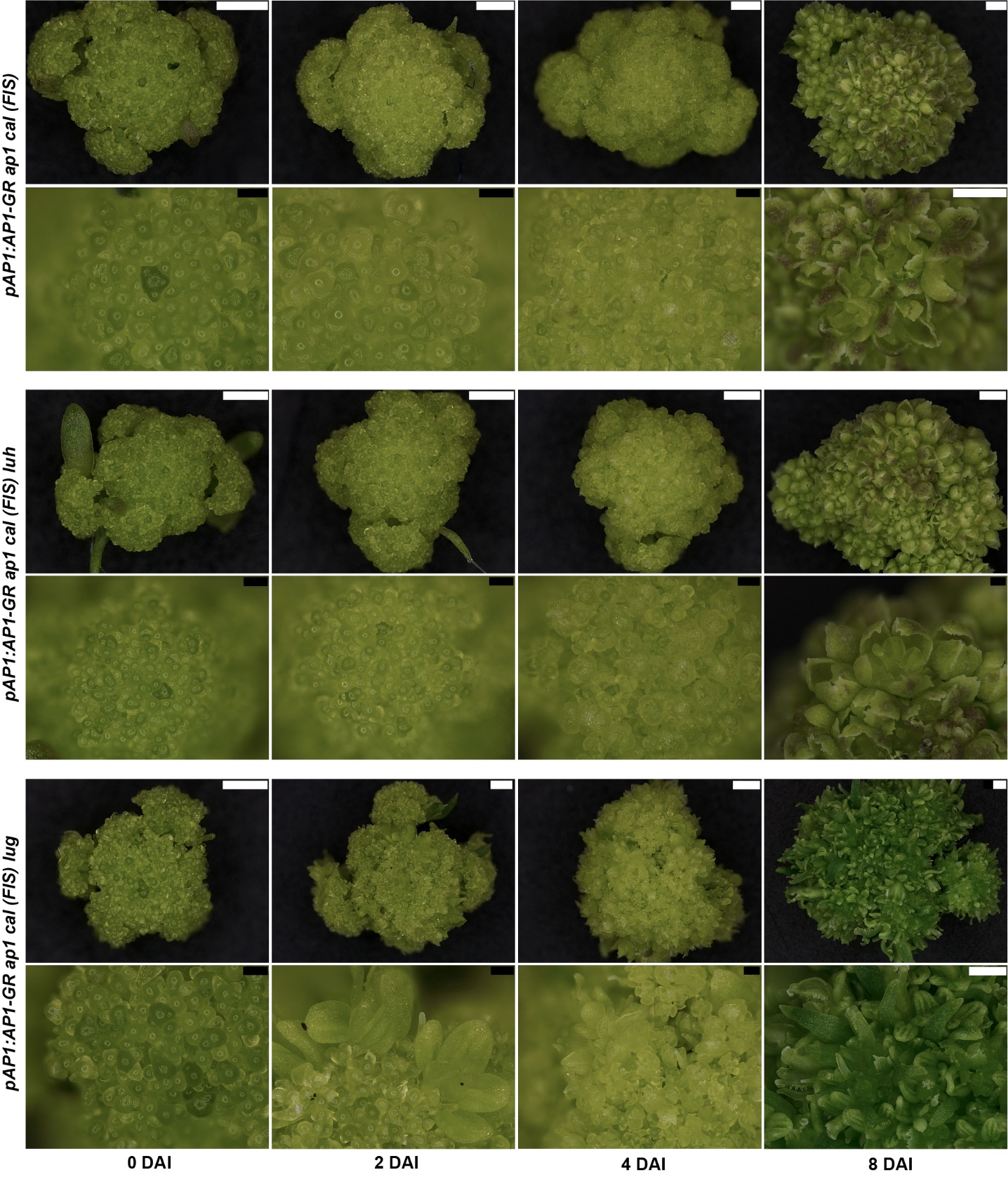


**Figure S10.** Phenotypic analysis of FIS, *luh* FIS, and *lug* FIS inflorescences at different stages of flower development. Scale bars are 500 µm (white) and 100 µm (black).


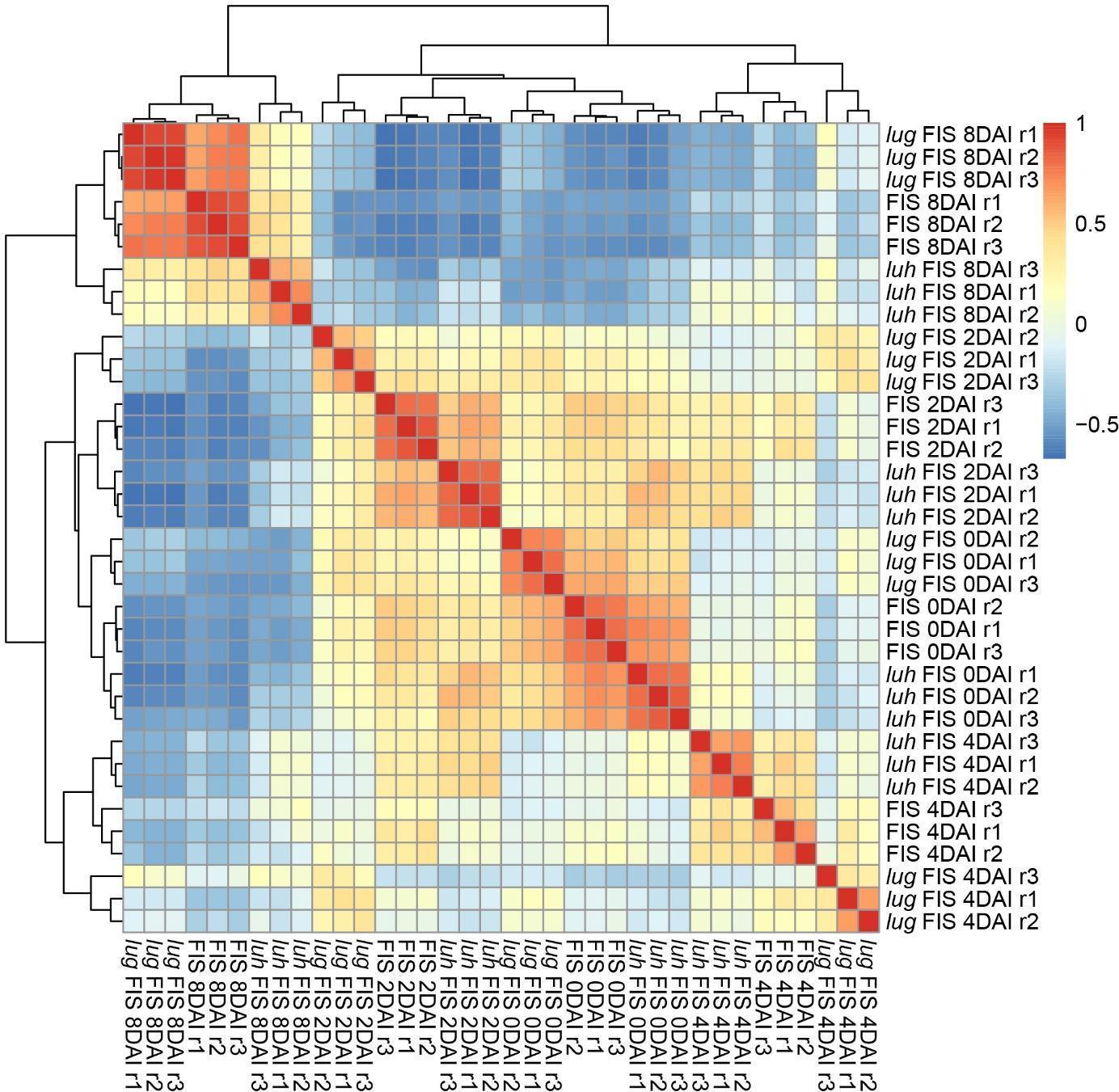


**Figure S11.** Reproducibility of relative expression values from independent biological replicates of FIS, *luh* FIS, and *lug* FIS inflorescences. Shown is a correlation heatmap based on the variance-stabilized normalized counts from DESeq2. Scale represents the Pearson correlation coefficient between pairs of samples after DESeq2 normalization.


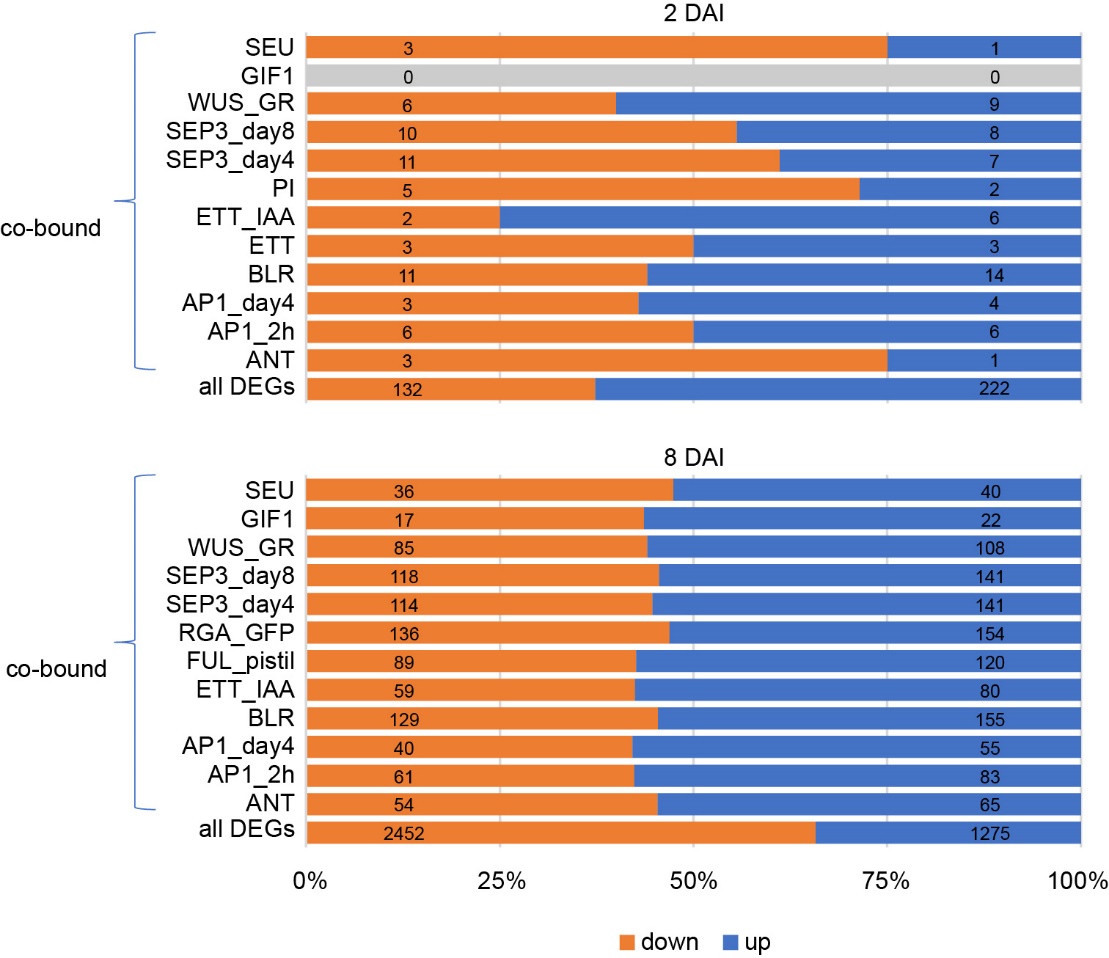


**Figure S12.** Gene expression analysis (*luh* FIS vs. FIS) of co-bound genes in LUH and other TF ChIP-seq across different stages of flower development. Time-points 0 DAI and 4 DAI data are not shown since the number of co-bound genes is lower than 5 for the majority of the ChIP-seq data. Numbers within bars indicate the number of DEGs.

**Supporting tables**

**Table S1.** Complementation of the *luh-5* seed mucilage defect by *proLUH:LUH-GFP*. Average mucilage thickness (µm) measured in seeds of Col-0, *luh-5*, and independent *proLUH:LUH-GFP luh-5* transgenic lines. Values represent the mean ± SEM. Statistical significance was assessed by two-tailed Student’s *t*-test compared to Col-0.

| Seeds | | Average mucilage thickness (µm) | SEM (µm) | *t*-test *p*-value vs Col-0 |
| --- | --- | --- | --- | --- |
| Col-0 | | 72.44 | 1.13 |  |
| *luh-5* | | 0.00 | 0.00 |  |
| *proLUH:LUH-GFP* in *luh-5* | line 9 | 70.37 | 2.03 | 4.07E-01 |
|  | line 5 | 55.90 | 2.39 | 8.57E-22 |
|  | line 2 | 54.79 | 1.78 | 1.10E-11 |
|  | line 8 | 53.90 | 3.24 | 9.35E-06 |
|  | line 12 | 50.05 | 2.39 | 8.28E-12 |
|  | line 7 | 30.95 | 2.06 | 1.00E-27 |
|  | line 11 | 24.62 | 1.53 | 1.94E-38 |
|  | line 3 | 24.50 | 1.51 | 1.64E-39 |
|  | line 6 | 21.98 | 2.11 | 1.36E-32 |
|  | line 10 | 18.90 | 2.78 | 1.72E-27 |
|  | line 16 | 17.60 | 1.70 | 4.74E-34 |
|  | line 15 | 8.90 | 2.08 | 1.59E-34 |
|  | line 14 | 5.81 | 1.25 | 1.41E-34 |
|  | line 4 | 1.00 | 0.54 | 2.09E-68 |
|  | line 13 | 0.20 | 0.20 | 5.19E-73 |
|  | line 1 | 0.00 | 0.00 | 1.80E-68 |

**Table S2.** Co-IP-MS proteomics data for LUH and AP1 proteins. Subset of MaxQuant-derived results (number of razor and unique peptides per protein and label-free quantification of protein intensities) for LUH and AP1 proteins detected in input and immunoprecipitation samples. These data correspond to the quantitative Co-IP-MS analysis shown in Figure 4d. ^†^ MaxQuant could not align/normalize peptides across runs.

| Co-IP-MS experiment | Razor + unique peptides | | LFQ intensity | |
| --- | --- | --- | --- | --- |
|  | LUH | AP1 | LUH | AP1 |
| LUH+AP1 Input | 27 | 17 | 5.33E+09 | 1.26E+10 |
| LUH+AP1 IP | 23 | 20 | 7.73E+09 | 1.94E+11 |
| LUH+empty Input | 33 | 2 | 1.67E+10 | 5.01E+07 |
| LUH+empty IP | 4 | 4 | 1.69E+09 | 1.28E+09 |
| LUH_LQ+AP1 Input | 16 | 18 | 9.04E+09 | 1.44E+10 |
| LUH_LQ+AP1 IP | 1 | 26 | 0^†^ | 2.52E+11 |
| LUH_LQ+empty Input | 17 | 1 | 1.77E+10 | 8.93E+07 |
| LUH_LQ+empty IP | 0 | 3 | 0 | 1.10E+09 |
| LUH_QW+AP1 Input | 25 | 17 | 6.75E+09 | 1.27E+10 |
| LUH_QW+AP1 IP | 20 | 18 | 1.03E+10 | 1.60E+11 |
| LUH_QW+empty Input | 29 | 3 | 1.31E+10 | 1.63E+08 |
| LUH_QW+empty IP | 3 | 5 | 1.31E+09 | 7.56E+08 |
| LUH_W+AP1 Input | 15 | 16 | 6.22E+09 | 1.17E+10 |
| LUH_W+AP1 IP | 10 | 25 | 2.86E+09 | 2.03E+11 |
| LUH_W+empty Input | 15 | 1 | 1.59E+10 | 1.68E+08 |
| LUH_W+empty IP | 2 | 3 | 6.93E+08 | 6.58E+08 |

**Table S3.** Number of significant peaks and their closest associated genes identified in ChIP-seq experiments. ^†^ Analysis performed using IDR correction with a threshold of 0.05.

| ChIP-seq dataset | *p*-value < 0.05 | | FDR < 0.05 | |
| --- | --- | --- | --- | --- |
|  | Significant peaks | Associated genes | Significant peaks | Associated genes |
| LUH-GFP FIS 0 DAI | 1044 | 879 | 0 | 0 |
| LUH-GFP FIS 2 DAI | 2944 | 2297 | 1018 | 843 |
| LUH-GFP FIS 4 DAI | 1790 | 1473 | 307 | 259 |
| LUH-GFP FIS 8 DAI | 4560 | 3315 | 2246 | 1752 |
| SEU-GFP *seu-1* |  |  | 1450^†^ | 1268^†^ |

**Table S4.** Number of differentially expressed genes identified in RNA-seq experiments.

| Sample  Stage | *luh* FIS vs FIS | | | *lug* FIS vs FIS | | | *luh* FIS vs *lug* FIS | | |
| --- | --- | --- | --- | --- | --- | --- | --- | --- | --- |
|  | Up | Down | Total | Up | Down | Total | Up | Down | Total |
| 0 DAI | 169 | 120 | 289 | 280 | 201 | 481 | 409 | 402 | 811 |
| 2 DAI | 222 | 132 | 354 | 570 | 421 | 991 | 661 | 644 | 1305 |
| 4 DAI | 119 | 127 | 246 | 542 | 295 | 837 | 420 | 665 | 1085 |
| 8 DAI | 1275 | 2452 | 3727 | 791 | 518 | 1309 | 1800 | 3209 | 5009 |

**Table S5.** Jaccard similarity indices for pairwise ChIP-seq experiment comparisons.

| Sample | LUH-GFP FIS 0 DAI | LUH-GFP FIS 2 DAI | LUH-GFP FIS 4 DAI | LUH-GFP FIS 8 DAI | SEU-GFP seu-1 |
| --- | --- | --- | --- | --- | --- |
| LUH-GFP FIS 0 DAI | 1.00 | 0.01 | 0.06 | 0.01 | 0.01 |
| LUH-GFP FIS 2 DAI | 0.01 | 1.00 | 0.38 | 0.46 | 0.16 |
| LUH-GFP FIS 4 DAI | 0.06 | 0.38 | 1.00 | 0.32 | 0.13 |
| LUH-GFP FIS 8 DAI | 0.01 | 0.46 | 0.32 | 1.00 | 0.17 |
| SEU-GFP seu-1 | 0.01 | 0.16 | 0.13 | 0.17 | 1.00 |

**Table S6.** Primers used for cloning *LUH*, *LUG* and *SEU* constructs. List of primers used to generate LUH and SEU full-length or domain-specific constructs in the pSPUTK vector as well as *proLUG:LUG(CDS)-GFP* construct. Restriction sites are underlined in the sequences.

| Primer | Sequence [5’-3’] |
| --- | --- |
| proLUG_SalI_F | ATCgtcgacTAAGCTAGTAGGGGATATGGAC |
| proLUG_ApaI_R | ATCgggcccCTCTGTATAGTTCTTTGGACA |
| LUG(CDS)_F | ATGTCTCAGACCAACTGGGA |
| LUG(CDS)_R | CTTCCACAGTTTCACTAGCTTATCA |
| LUH_NcoI_F | AATccATGGCTCAGAGTAATTGGGA |
| LUH_ClaI_R | AATatcgatCTACTTCCAAATCTTTACGGATTTGT |
| LUH_Q_NcoI_F | CCGGccATGGGTAAAGCGAAGGAGCA |
| LUH_Q_ ClaI_R | CCGGatcgatCTAGGCGGCATGCTGATATG |
| LUH_W_ NcoI_F | CCGGccATGGTACAGCAATCTTCTTCTCAGC |
| SEU_ApaI_F | ATCgggcccATGGTACCATCAGAGCCGC |
| SEU_ClaI_R | AATatcgatTCACGCGTTCCAATCAAAATTGT |
| SEU_D_ClaI_R | CCGGatcgatTCATAAACTCTCGATTGGTCC |
| SEU_Q1_ApaI_F | CCGGgggccCAGGTTTCGAATGATCAGCA |
| SEU_D_ApaI_F | CCGGgggccCATAGGCCTGAAGACAATAA |
